# Polyfunctionality of CD4^+^ T lymphocytes in buffaloes and cattle: comparative antigen-specific cytokine responses in bovine tuberculosis infection

**DOI:** 10.3389/fimmu.2025.1608065

**Published:** 2025-09-08

**Authors:** Susana Flores-Villalva, Giovanna De Matteis, Francesco Grandoni, Maria Carmela Scatà, Anna Donniacuo, Lorena Schiavo, Giulia Franzoni, Piera Mazzone, Mahmoud Elnaggar, Esterina De Carlo, Giorgio Galiero, William C. Davis, Alessandra Martucciello

**Affiliations:** ^1^ Centro Nacional de Investigacion Disciplinaria en Salud Animal e Inocuidad, Instituto Nacional de Investigaciones Forestales, Agricolas y Pecuarias (INIFAP), Mexico City, Mexico; ^2^ Consiglio per la ricerca in agricoltura e l’analisi dell’economia agraria (CREA)-Animal Production and Aquaculture, Monterotondo, Italy; ^3^ National Reference Centre for Hygiene and Technologies of Water Buffalo Farming and Productions, Istituto Zooprofilattico Sperimentale del Mezzogiorno, Salerno, Italy; ^4^ Department of Animal Health, Istituto Zooprofilattico Sperimentale della Sardegna, Sassari, Italy; ^5^ Centro Specialistico di Ricerca Applicata alle Micobatteriosi, Istituto Zooprofilattico Sperimentale dell’Umbria e delle Marche, Perugia, Italy; ^6^ Department of Veterinary Medicine, College of Applied and Health Sciences, A’Sharqiyah University, Ibra, Oman; ^7^ Department of Microbiology, Faculty of Veterinary Medicine, Alexandria University, Egypt; ^8^ Department of Veterinary Microbiology and Pathology, Washington State University, Pullman, WA, United States

**Keywords:** flow cytometry, tuberculosis, cytokines, polyfunctional lymphocytes, cattle, buffalo

## Abstract

**Introduction:**

Bovine tuberculosis (BTB), caused by *Mycobacterium bovis*, is a chronic infectious disease of major veterinary and public health concern. It affects a broad range of domestic and wild animals, including water buffalo, and poses a risk to humans due to its zoonotic nature. The economic consequences of BTB, arising from production losses and trade restrictions, further underline its global importance. While cattle immune responses to BTB are well characterized, the immune mechanisms in buffalo remain poorly understood, despite their increasing role as livestock in endemic regions. Given that buffaloes and cattle, although closely related, display notable immunological differences, comparative studies are essential. This study aimed to investigate and compare antigen-specific cytokine responses in CD4^+^ T lymphocytes from buffaloes and cattle exposed to or infected with *M. bovis*.

**Methods:**

A multicolor flow cytometry assay was established to enable high-resolution analysis of cytokine-expressing CD4^+^ T cells. Blood samples were obtained from 35 buffaloes (17 IGRA-positive from BTB outbreak farms and 18 IGRA-negative, including animals from both outbreak and Officially Tuberculosis-Free [OTF] herds) and 10 cattle (6 IGRA-positive from a BTB outbreak farm and 4 IGRA-negative from an OTF herd). Following six hours of in vitro stimulation with PPD-B or PBS, intracellular cytokine staining was performed. This approach allowed simultaneous quantification of single and polyfunctional CD4^+^ T cell subsets producing IFN-γ, TNF-α, and IL-17A. Data were analyzed using factor analysis of mixed data (FAMD) to explore species- and infection-related immune response patterns.

**Results:**

The multicolor flow cytometry approach successfully identified distinct cytokine-producing CD4⁺ T cell populations in both species. Overlapping immune profiles were observed between buffaloes and cattle; however, specific subsets—including IL-17A^+^, IFN-γ^+^IL-17A^+^, and TNF-α^+^IL-17A^+^ cells—contributed to interspecies differences. Importantly, the frequency of IFN-γ^+^ and TNF-α^+^ producing CD4^+^ T cells correlated with IGRA test status, enabling discrimination between infected/exposed and non-infected animals. These results demonstrate the ability of cytokine expression patterns to reflect both infection status and host species.

**Discussion:**

The findings indicate that buffaloes and cattle share broadly similar antigen-specific cytokine responses, although subtle differences in CD4⁺ T cell subsets exist. The study highlights the value of multicolor flow cytometry as a high-resolution tool for dissecting immune responses in veterinary immunology. These insights enhance understanding of buffalo immune mechanisms against BTB and may contribute to improved disease control strategies.

## 1 Introduction

Bovine tuberculosis (BTB), caused by *Mycobacterium tuberculosis complex* (MTBC), primarily *M. bovis* and *M. caprae*, remains a significant economic and public health concern, particularly in developing countries and endemic wildlife areas. MTBC infection affects a wide range of animals, including ruminants such as cattle and buffaloes (1–3). Although both species belong to the Bovidae family, they exhibit notable anatomical and physiological differences, including variation in disease susceptibility (4–6).

The immune response to mycobacterial infections involves a coordinated effort between innate and adaptive immune cells. Among this, CD4^+^ T lymphocytes are key players in defending against tuberculosis (TB), mainly by producing interferon-gamma (IFN-γ), a cytokine critical for activating macrophages and controlling infection by MTBC (7, 8). Additionally, CD4^+^ T cells secrete other important cytokines such as interleukin-2 (IL - 2), tumor necrosis factor-alpha (TNF-α), and interleukin-17 (IL - 17) which help recruit immune cells to the infection site and supports the development of specialized CD4^+^ T cell subsets capable of performing effector functions (9). Polyfunctional CD4^+^ T cells, which can simultaneously produce multiple cytokines, have been found in peripheral blood of individuals with TB (10). However, the role of these polyfunctional CD4^+^ T cells play controlling the infection remains unclear, with studies showing conflicting results. Some reports have found a higher frequency of triple-producing CD4^+^ T cells (IFN-γ, IL - 2, and TNF-α) in patients with active TB disease in comparison to patients with latent TB-infection. While, single (IFN-γ) or dual-producing CD4^+^ T cells (IL - 2, IFN-γ) were higher in cured TB patients and patients with latent TB (10). On the other hand, some authors have reported that smear-negative TB patients had higher frequencies of polyfunctional CD4^+^ T than smear-positive TB patients, and therefore the presence of polyfunctional CD4^+^ T are associated with a progressive T cell dysfunction and high mycobacterial loads (11).

Research on polyfunctional CD4^+^ T cells in cattle is limited. However, one study reported a higher frequency of triple producing CD4^+^ T (IFN-γ, IL - 2, TNF-α) in naturally BTB-infected cattle compared to uninfected animals. These cells exhibited a T-effector memory phenotype and expressed more cytokine than single producing CD4^+^ T cells, suggesting an association with active disease (12). Moreover, polyfunctional CD4^+^ T cells have been proposed as potential correlates of protection in TB vaccine studies (13). A higher frequency of triple producing CD4^+^ T cells has been associated with a protective response to mycobacterial infections in murine, bovine and non-human primate TB models using BCG and new TB vaccine candidates (13, 14). Despite these findings, the role of polyfunctional CD4^+^ T in mycobacterial immunity remains unclear in both humans and animals. Interestingly, CD4^+^ and γδ T cells have been identified as the primary producers of IL - 22 and IL - 17A in lymphocytes from *Mycobacterium bovis*-infected cattle, underscoring the potential significance of IL - 17A-producing T cells in the immune response to BTB. This finding highlights their possible role in promoting inflammatory processes and contributing to protective immunity in infected cattle (15). Furthermore, a study by Elnaggar M. et al., characterized αβ and γδ T cell subsets expressing IL - 17A in ruminants and swine, reinforcing the importance of IL - 17A-producing T cells as key players in the immune response to mycobacterial infections (16). Together, these studies provide valuable insight into the complex interplay between T cell subsets and cytokine production in response to *M. bovis* infection.

Buffaloes plays a pivotal role in the economy of many countries; due to its rusticity and versatility these animals are being increasingly recognized as a production and commercial option under harsh climate conditions (17). Buffaloes can be affected by the same diseases as cattle; although it seems that BTB in buffalo progress at lower pace in comparison to cattle, with animals having no gross lesions in the first year of infection (18). Since few studies have investigated the immune response to *Mycobacterium bovis* in buffaloes and cattle (19, 20) our study focused on the role of polyfunctional CD4^+^ T cells producing IFN-γ, TNF-α, and IL - 17A in both healthy and naturally infected Mediterranean water buffalo (*Bubalis bubalis*). To deepen our understanding of species-specific immune responses, we conducted a comparative analysis with cattle. These findings offer valuable insights into the pathogenesis of bovine tuberculosis (BTB) and may inform the development of tailored diagnostic tools, vaccines, and control strategies for both species. Finally, the identification of key differences in immune responses between buffaloes and cattle may help reduce disease burden and cross-species transmission in regions where both species coexist.

## 2 Materials and methods

### 2.1 Animals and study design

Italian Mediterranean buffaloes used in this study were analyzed as part of the officially mandated TB surveillance program in Italy, following national (DM 592/95; D Lgs 196/1999; Regulation (EU) 2016/429 ‘Animal Health Law’, which replaced Commission Regulation EC 1226/2002; O. M. 9 August 2012, and subsequent amendments) and regional (DGRC 108 104/2022) regulations. The bovine tuberculosis (BTB) status of animals was determined using ante-mortem single intradermal tuberculin (SIT) test and IFN-γ release assay (IGRA), in accordance with official guidelines. IFN-γ test was carried out by Istituto Zooprofilattico Sperimentale del Mezzogiorno (IZSME) Italy (see 2.2). SIT and IGRA positive reactors were slaughtered in accordance with national and regional legislation and then the presence of TB-like lesions was evaluated. The organs were sent to the IZSME for investigation of the presence of *M. bovis* using PCR for *M. bovis* DNA detection and culture isolation of MTBC mycobacteria, according to the World Organization for Animal Health (WOAH) Terrestrial Manual (21). Buffaloes from the BTB outbreak farms (except one) were negative for lesions and PCR for the detection of *M. bovis* DNA. Whereas all cattle from the BTB outbreak farms were positive for BTB-like lesions and PCR of *M. bovis*.

A total of 35 buffaloes and 10 cattle were enrolled in the study and classified according to their IGRA test results: positives animals (17 buffaloes and 6 cattle) and negatives animals (18 buffaloes and 4 cattle). Naturally exposed buffaloes were selected from two herds with confirmed BTB outbreaks by the isolation of *M. bovis.* Naturally BTB-infected cattle were selected from a herd with confirmed BTB outbreaks, while uninfected animals were sourced from Officially Tuberculosis-Free (OTF) herds in the Campania region (Italy). OTF herds were selected based on negative results from the annual SIT test and IGRA test screenings conducted over the last six years.

The IGRA positive buffaloes had a mean age of 2.25 ± 1.48 years, while the negative had a mean age of 7.35 ± 0.49 years. Cattle were selected from one BTB positive farm and one OTF herd. IGRA positive cattle had a mean age of 6.04 ± 0.05 and negative of 5.95 ± 0.07 years.

Additionally, buffaloes were divided into three groups based on results of the IGRA test and the status of the herd. Group A) IGRA positive animals from BTB outbreak farm (n=17); Group B) IGRA negative animals from BTB outbreak farm (n=13); Group C) IGRA negative animals from OTF farm (n=5). This approach allowed us to analyze cytokine production in IGRA negative buffaloes that had been in contact with IGRA positive animals, helping to identify potential differences in immune responses.

### 2.2 Interferon gamma test (IGRA)

The IFN-γ test was performed using blood samples collected from the jugular vein in lithium-heparin vacutainer tubes (BD Biosciences) and transported to the laboratory within 8 h of collection. Two aliquots of blood (1 mL) per animal were incubated with 10 μg/mL of avian and bovine purified protein derivative, respectively (PPD-A and PPD-B BOVIGAM™ TB kit Thermo-Fisher Scientific). Additionally, two aliquots of blood (1 mL) per animal were stimulated with pokeweed mitogen (PWM, final concentration 1 μg/mL, Thermo-Fisher Scientific) as positive control, and phosphate buffered saline (PBS) as baseline and negative control. The samples were incubated for 16 – 24 hours at 37°C in a humidified atmosphere. IFN-γ levels were quantified using the BOVIGAM™ test, following the manufacturer’s instructions (Life Technologies, Thermo-Fisher Scientific). A sample was considered positive when the differences of the optical density (OD) of PPDB–PPDA and PPDB-PBS were ≥0.1 OD (22). IGRA results from all animals used in this study are reported in **Supplementary Figure S1**.

### 2.3 Whole blood stimulation for flow cytometry assay

Blood samples were processed within 24 hours of collection. The protocol for blood stimulation was carried out as described by De Matteis et al., with some modifications (19). One milliliter of blood was dispensed into 5 mL polystyrene round bottom tubes (Sarstedt) and stimulated with PPD-B, PWM and PBS (same concentrations used for the IGRA test). PWM and PBS were used as positive and negative controls respectively. All tubes were incubated at 37°C for 2 h in a humidified atmosphere with 5% CO_2_. After 2 h of incubation, brefeldin A (10 μg, Thermo-Fisher Scientific) was added and the samples were incubated for further 4 h.

### 2.4 Flow cytometry assay

To assess the cross-reactivity of anti-bovine TNF-α and IL - 17A antibodies with buffalo antigens, a preliminary study was conducted using Peripheral Blood Mononuclear Cells (PBMCs). PBMCs were isolated from EDTA-anticoagulated blood via density gradient centrifugation using Lymphoprep™ (1.077 g/mL; AXIS-SHIELD), following the manufacturer’s instructions. The isolated cells were washed with PBS (without Ca^2+^/Mg^2+^), centrifugated at 300 x g for 10 minutes, counted, and cultured in complete RPMI medium at a concentration of 1×10^6^ cells/mL. Then, cells were stimulated for 4 hours with PMA (phorbol 12-myristate 13-acetate; Cell Stimulation Reagent with Brefeldin A, Bio-Rad). Following stimulation, cells were washed twice with PBS, resuspended in 125 μL of fixation/permeabilization buffer (Cytofix/Cytoperm™, BD Biosciences), and incubated at 4°C for 20 minutes. After two washes with 800 μL of Perm/Wash buffer (PWB), the cells were centrifuged at 600 × g for 10 minutes. The permeabilized cells were then resuspended in 100 μL of PWB and incubated at 4°C for 30 minutes with 0.75 μg/5 μL of anti-TNF-α or anti-IL-17A monoclonal antibodies. Subsequently, 1 mL of PWB was added to the cells, followed by centrifugation at 600 × g for 5 minutes. The resulting pellet was resuspended in 50 μL of a 1:200 dilution of goat anti-mouse IgG1 FITC-conjugated secondary antibody (Bio-Rad) and incubated for 15 minutes at 4°C in the dark. After a final wash with PWB, the stained cells were collected using a CytoFLEX flow cytometer and analyzed with CytExpert v2.4 software (Beckman Coulter).

For the study, a four-color panel was developed to evaluate the intracellular cytokine expression of IFN-γ, IL - 17A and TNF-α within CD4^+^ T lymphocytes (**Supplementary Table S1**).

100 μL of stimulated whole blood (section 2.3) was lysed with 1 mL of Tris-buffered ammonium chloride solution (0.87% w/v, pH 7.3) for 10 min, washed once with 2 mL of cold PBS and then incubated at 4°C for 30 min with anti-bovine CD4^+^ labeled in-house with PE-Cy7 (Lynx technology, Bio-Rad). After staining, the leukocytes were washed with 2 mL of cold PBS and centrifuged at 300 × g for 5 min. The pellets were resuspended in 125 μL of PWB (Cytofix/Cytoperm™ solution, BD Biosciences) and incubated at 4°C for 20 min. After two cycles of washing with 800 μL of PWB and centrifugations at 600 × g for 10 min, the permeabilized cells were incubated at 4 °C for 30 min with the following in-house labeled (Lynx technology, Bio-Rad) antibodies: anti-TNF-α FITC, anti-IL-17A PE, and anti-IFN-γ AF647. After a wash with 800 μL of PWB and centrifugation at 600 × g for 5 min, the cells were resuspended in the same buffer and collected on a CytoFLEX flow cytometer (Beckman Coulter). An average of 5,000 events were collected within the gate of CD4^+^ cells. Kaluza software (Beckman Coulter) was used to analyze flow cytometry data. Gating strategy was applied to identify CD4^+^ T cell subsets based on cytokine co-expression profiles. Single cytokine-producing cells refer to CD4^+^ T cells that express only one cytokine (IFN-γ, TNF-α, or IL - 17A) without co-expression of the others, whereas dual cytokine-producing cells co-express two cytokines (IFN-γ^+^IL-17A^+^, IFN-γ^+^TNF-α^+^, TNF-α^+^IL-17A^+^). The analysis of the frequency of reactive cells was performed after the subtraction of cells stimulated with PBS.

### 2.5 Statistical analysis

The data analysis was conducted on R (version 4.4.1) using R studio (version 2024.04.2). The Factor analysis of mixed data (FAMD) was performed using the FactoMineR, factoextra, ggplot2 and tidyverse packages. Comparisons between groups (IGRA positive vs IGRA negative animals) and (buffaloes vs cattle) were analyzed using Wilcoxon text with Bonferroni correction using the tidyverse, rstatix, and ggpubr packages. The spider plots graphs were generated with the mean scaled data using the ggradar package. Pearson correlation analysis was performed to analyze the relationship between IGRA results (OD PPDB - OD PBS) and the different cell subsets using the Hmisc package. Finally, box plots were designed using GraphPad Prism 8 with data expressed as mean ± SD in all parameters.

## 3 Results

### 3.1 Flow cytometric evaluation of cytokines

The cross-reactivity of anti-bovine TNF-α and IL - 17A monoclonal antibodies in water buffalo was preliminarily assessed through cytokine staining in PMA-stimulated PBMCs from both species, which revealed similar expression patterns (**Supplementary Figure S2**) (23).

In this study, a 6-hour whole blood stimulation assay was performed to assess the production of single (IFN-γ^+^, IL - 17A^+^, TNF-α^+^) and dual-producing (IFN-γ^+^IL-17A^+^, IFN-γ^+^TNF-α^+^, TNF-α^+^IL-17A^+^) CD4^+^ lymphocytes in buffalo and cattle. Furthermore, the gating strategy allowed for the identification of the following CD4^+^ T cell subsets, organized for clarity and diagnostic relevance: IFN-γ^+^TNF-α^neg^, IFN-γ^+^IL-17A^neg^, IFN-γ^neg^TNF-α^+^, IFN-γ^neg^IL-17A^+^, TNF-α^+^IL-17A^neg^, and TNF-α^neg^IL-17A^+^. This approach helped resolve overlapping populations and better interpret the functional identity of each subset within the same multiparametric dataset.

The gating strategy and the Fluorescence Minus One (FMO) controls for cytokines analysis are shown in **Supplementary Figure S3**.

**Figure 1:**
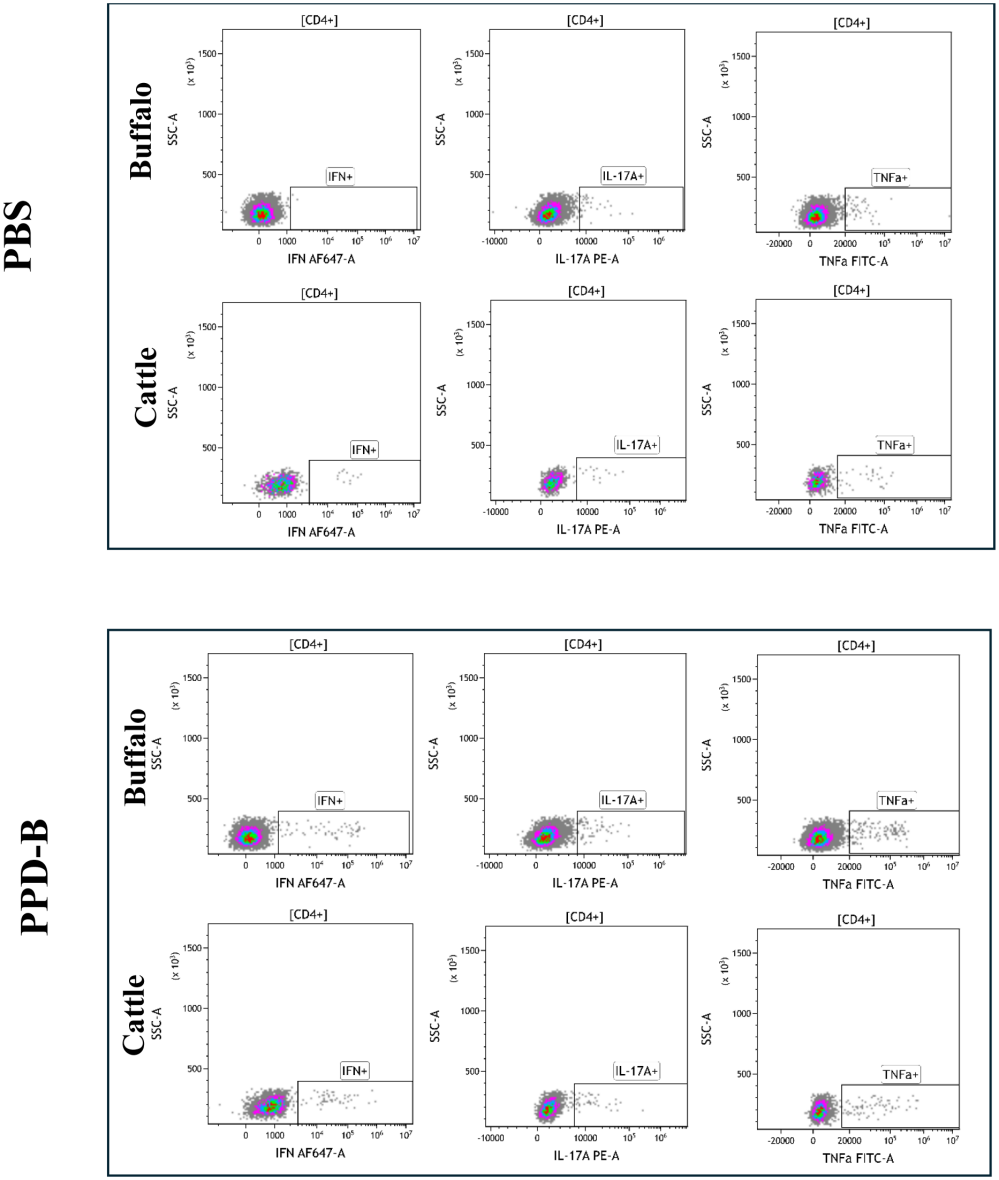
Single cytokine-producing CD4^+^ lymphocytes (IFN-γ^+^, IL - 17A^+^, TNF-α^+^) in buffalo and cattle after stimulation with PBS and PPD-B. Gates were drawn based on the Florescence Minus One control (FMO) for each cytokine in buffalo and in cattle.

**Figure 2:**
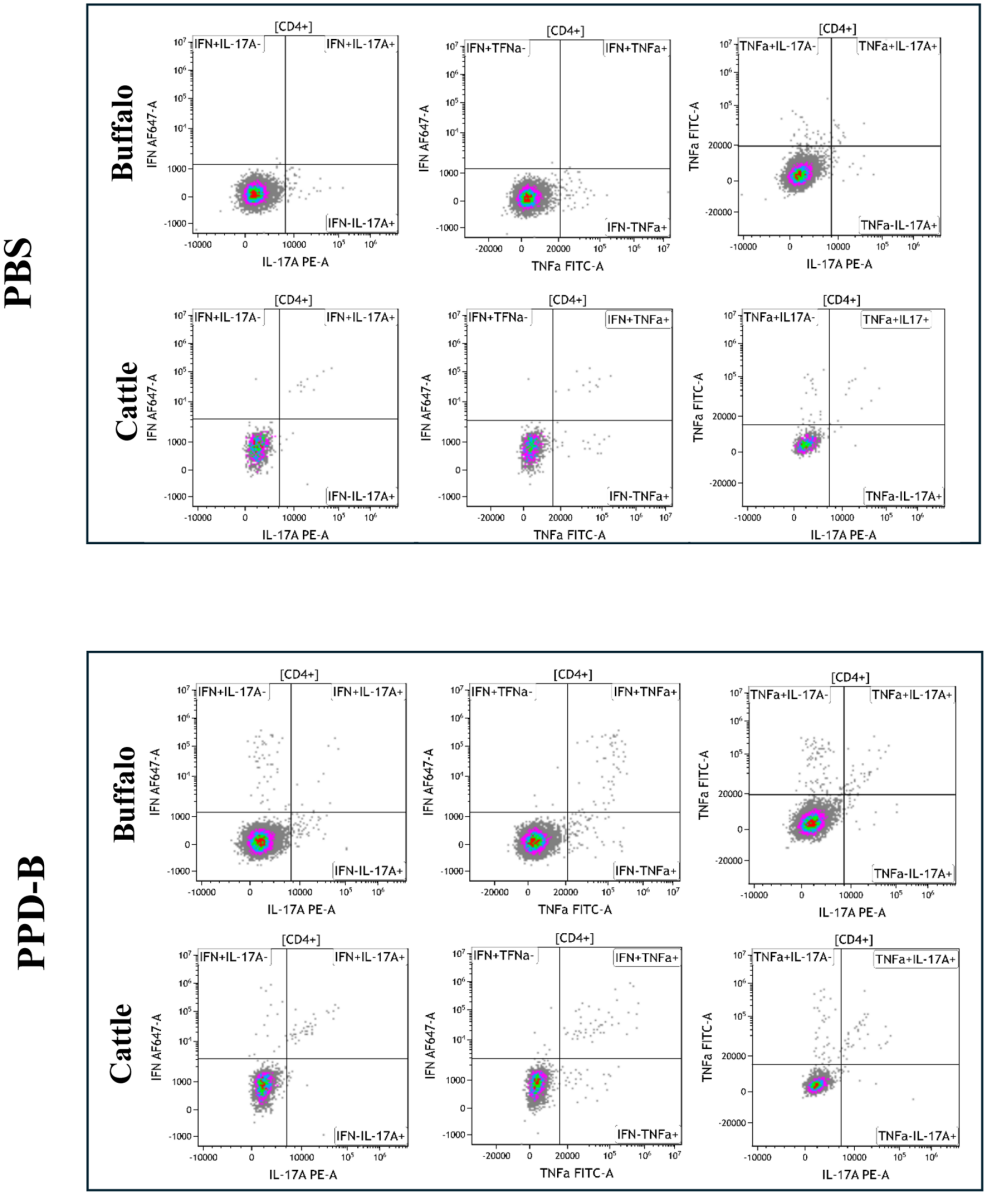
Dual cytokine-producing CD4^+^ lymphocytes (IFN-γ^+^TNF-α^+^, IFN-γ^+^IL-17A^+^, TNF-α^+^IL-17A^+^) and CD4^+^ subsets (IFN-γ^+^IL17A^neg^, IFN-γ^+^TFN-α^neg^, IFN-γ^neg^TNF-α^+^, IFN-γ^neg^IL17A^+^, TNF-α^+^IL17A^neg^, TNF-α^neg^IL17A^+^), in buffalo and cattle after stimulation with PBS and PPD-B. Quadrants were drawn based on the Florescence Minus One control (FMO) for each cytokine in buffalo and in cattle.

The cytokine staining patterns observed in buffalo and cattle following PBS and PPD-B stimulation are presented in **Figures 1**, **2**. PWM stimulation was used as a positive control to assess the overall capacity of CD4^+^ T cells to produce cytokines. PWM induced a strong cytokine response in both buffalo and cattle, with markedly increased frequencies of IFN-γ^+^, TNF-α^+^, and IL - 17A^+^ CD4^+^ T cells compared to unstimulated controls. This robust cytokine production confirms the functionality and responsiveness of T cells in the tested animals and validates the flow cytometry assay for detecting cytokine-expressing subsets. The consistent responses across species also support the cross-reactivity of the monoclonal antibodies used. Results from PWM stimulation, used as positive control, are provided in **Supplementary Figure S4**.

### 3.2 Frequencies of single and dual cytokine-producing CD4^+^ T cells in buffalo and cattle

**Figure 3:**
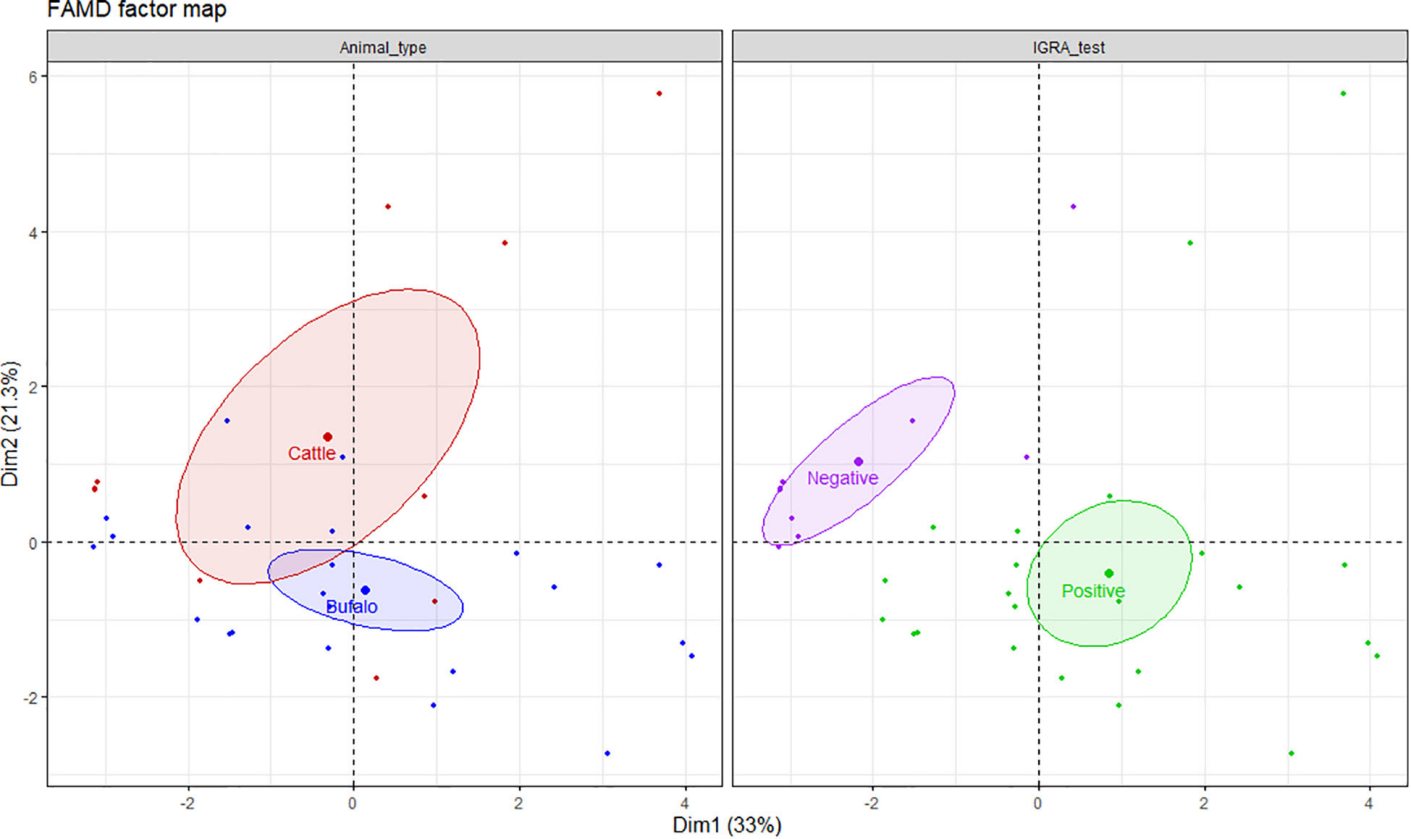
FAMD factor map based on single and dual cytokine-producing CD4^+^ T cells in buffalo and cattle. The left panel shows clustering by animal type (cattle in red, buffalo in blue), and the right panel by IGRA test result (positive in green, negative in purple). Ellipses represent 95% confidence intervals. Dim1 and Dim2 explain 33% and 21.3% of the variance, respectively, indicating distinct immunological profiles by species and IGRA test results. A total of 17 IGRA-positive and 5 IGRA-negative buffaloes, as well as 6 IGRA-positive and 4 IGRA-negative cattle, were included in the analysis.

The frequencies of single and dual cytokine-producing CD4^+^ T lymphocytes were investigated in buffalo and cattle in relation to positive or negative result of the IGRA test. To explore the relationship between the variables, a factor analysis of mixed data (FAMD) was performed. This principal component method allowed us to analyze both quantitative (cell frequencies) and qualitative variables (animal type, IGRA test result) (24). Overall, the FAMD plot shows the first two principal dimensions (Dim1 and Dim2), which together explain 54.3% of the variance in the dataset (Dim1: 33% and Dim2: 21.3%, respectively). Dim1 is represented mostly by the contribution of IFN-γ^+^, TNF-α^+^, IFN-γ^+^ TNF-α^+^, IFN-γ^+^ IL-17A^neg^ and TNF-α^+^ IL-17A^neg^ CD4^+^ T cells, whereas IL - 17A^+^, IFN-γ^+^ IL-17A^+^, IFN-γ^+^ IL-17A^neg^, IFN-γ^neg^ TNF-α^+^, TNF-α^+^ IL-17A^+^ CD4^+^ lymphocytes contribute most strongly to Dim2 (**Supplementary Table S2**). It is important to note that in FAMD, variables can contribute to multiple dimensions, as each dimension reflects a different latent structure or pattern of variation within the dataset. For example, IFN-γ^+^IL-17A^neg^ CD4^+^ cells occupy the fourth position in terms of contribution to both Dim1 and Dim2, indicating their relevance across distinct aspects of immune variability. The left panel of the FAMD plot shows that although there is some overlap in the frequencies of the cell subsets, Dim2 helps differentiate between cattle and buffalo. The size of the ellipse shows that cattle data exhibit greater variability along Dim2, whereas buffalo responses show less internal variation. The right panel shows that Dim1 clearly separates IGRA negative from IGRA positive animals indicating differences in immune profiles based on infection status. These results suggest that Dim1 primarily captures IFN-γ-dominant responses, while Dim2 reflects IL - 17A-associated immune variability, further highlighting the complexity and multidimensional nature of the cytokine response in both species (**Figure 3**).

**Figure 4:**
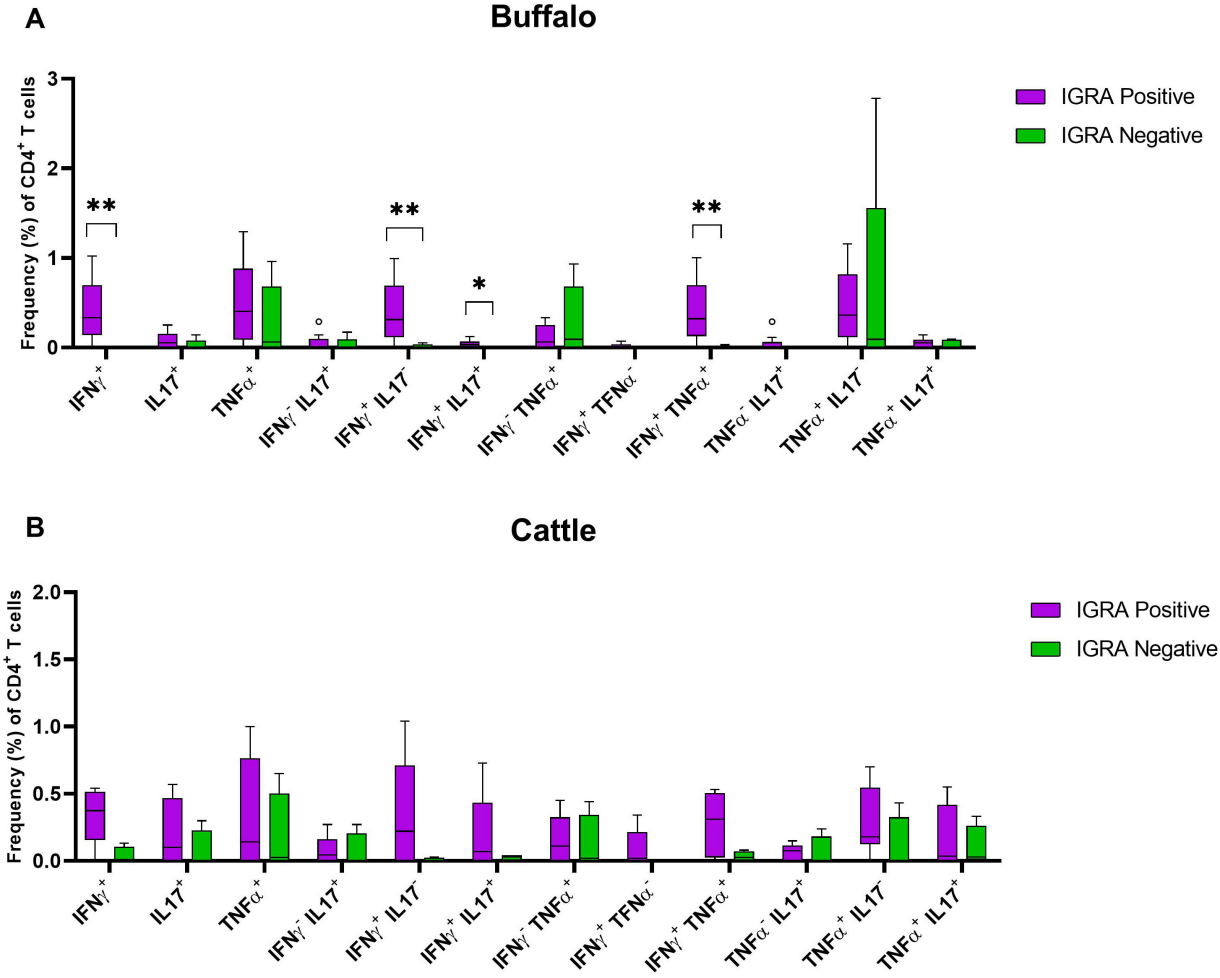
Frequencies (%) of single and dual-producing CD4^+^ T subsets of IFN-γ, TNF-α and IL - 17A in IGRA negative and positive animals. **(A)** Boxplots of the defined single and dual-producing CD4^+^ T subsets in buffalo. **(B)** Boxplots of the defined single and dual-producing CD4^+^ T subsets in cattle. Frequencies (%) of reactive cells are displayed after subtraction of cells stimulated with PBS. A total of 17 IGRA-positive and 5 IGRA-negative buffaloes, as well as 6 IGRA-positive and 4 IGRA-negative cattle, were included in the analysis. Results were compared using Wilcoxon test with Bonferroni correction. *P < 0.05; **P < 0.001.

**Figure 5:**
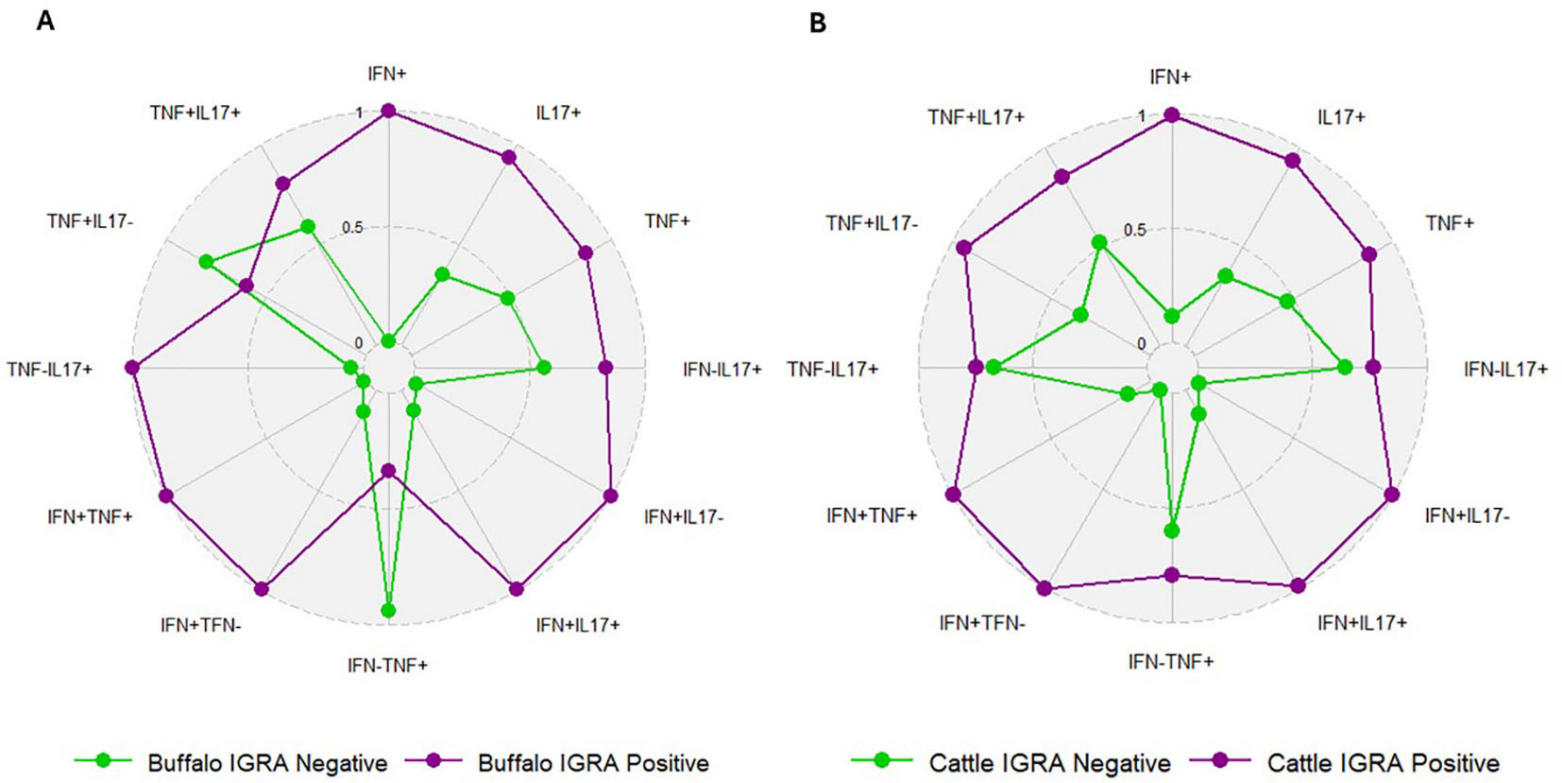
Spider plots based on data from **Figure 4**, representing the frequencies of single and dual cytokine-producing CD4^+^ T subsets in IGRA-negative and IGRA-positive buffaloes **(A)** and cattle **(B)**. The plots show similar patterns between species, with higher frequencies in IGRA-positive animals compared to IGRA-negative ones.

We compared the frequencies of single and dual cytokine-producing CD4^+^ T cell between IGRA positive and IGRA negative buffaloes (**Figure 4A**) and cattle (**Figure 4B**), respectively. The analysis revealed significantly higher frequencies of IFN-γ^+^, IFN-γ^+^ TNF-α^+^, IFN-γ^+^IL-17A^+^ and IFN-γ^+^IL-17A^neg^ CD4^+^ T cell subsets in IGRA positive buffalo compared to IGRA negative buffalos (**Figure 4A**). In cattle, although a similar trend was observed, no significant differences were detected between IGRA positive and IGRA negative cattle (**Figure 4B**) (**Supplementary Table S3**). The spider plots in **Figure 5** shows similar patterns in the frequencies of cytokines-positive CD4^+^ T cell subsets between buffalo and cattle, with higher number of cells in IGRA positive animals in comparison to IGRA negative ones.

**Figure 6:**
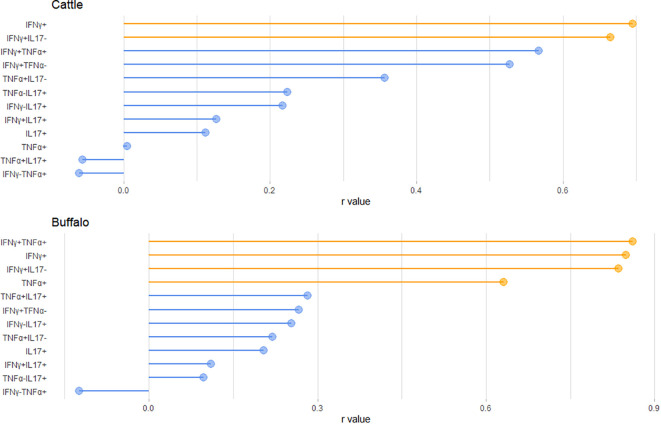
Lollipop plot showing the Pearson correlation coefficients (r-values) between the IGRA test result (PPDB-PBS) and the frequency of the indicated CD4^+^ T cell subsets in cattle (top) and buffalo (bottom). Variables highlighted in orange indicate statistically significant correlations (P < 0.05).

Our results suggest that the frequency of single and dual cytokine-producing CD4^+^ T cells varied according to the IGRA test result. In fact, correlation analysis between IGRA test results and the proportions of cytokine-producing CD4^+^ T cells following PPD-B stimulation revealed strong positive correlations in buffalo for the IFNγ^+^, TNF-α^+^, IFNγ^+^TNF-α^+^ and IFNγ^+^IL-17A^neg^ subsets (r = 0.8486, 0.6318, 0.8351, and 0.8612, respectively). Whereas, in cattle correlations were observed for the IFNγ^+^ and IFNγ^+^IL-17A^neg^ subsets (r = 0.6955 and 0.6647, respectively) (**Figure 6**).

**Figure 7:**
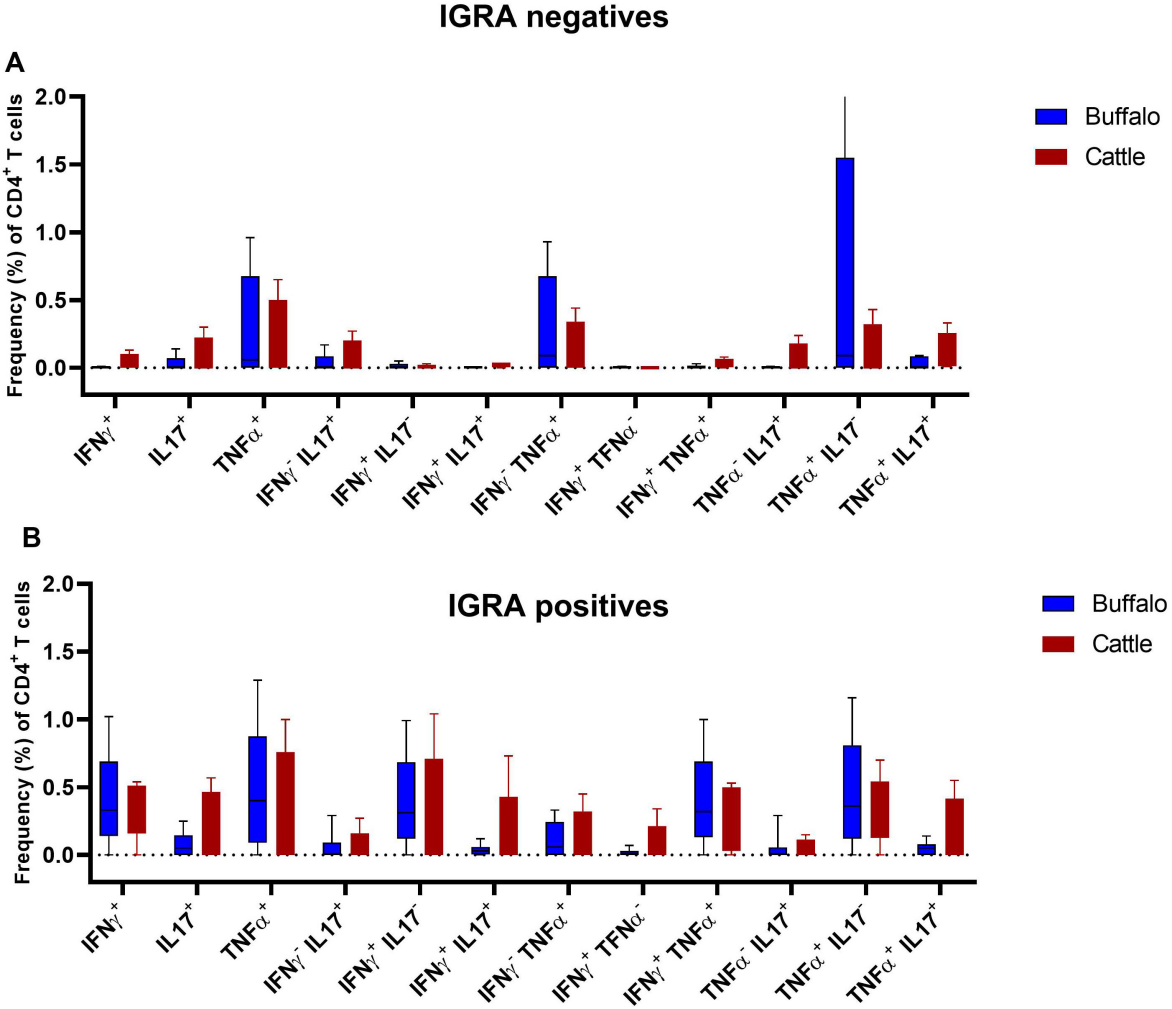
Frequencies (%) of single and dual cytokine-producing CD4^+^ T subsets of IFN-γ, TNF-α and IL - 17A in buffalo and cattle. **(A)** Boxplots of the defined single and dual producing CD4^+^ T subsets in IGRA negative animals. **(B)** Boxplots of the defined single and dual producing CD4^+^ T subsets in IGRA positive animals. Frequencies (%) of reactive cells are displayed after subtraction of cells stimulated with PBS. A total of 17 IGRA-positive and 5 IGRA-negative buffaloes, as well as 6 IGRA-positive and 4 IGRA-negative cattle, were included in the analysis. Results were compared using Wilcoxon test with Bonferroni correction. No statistical differences were found as showed in **Supplementary Table S4**.

**Figure 8:**
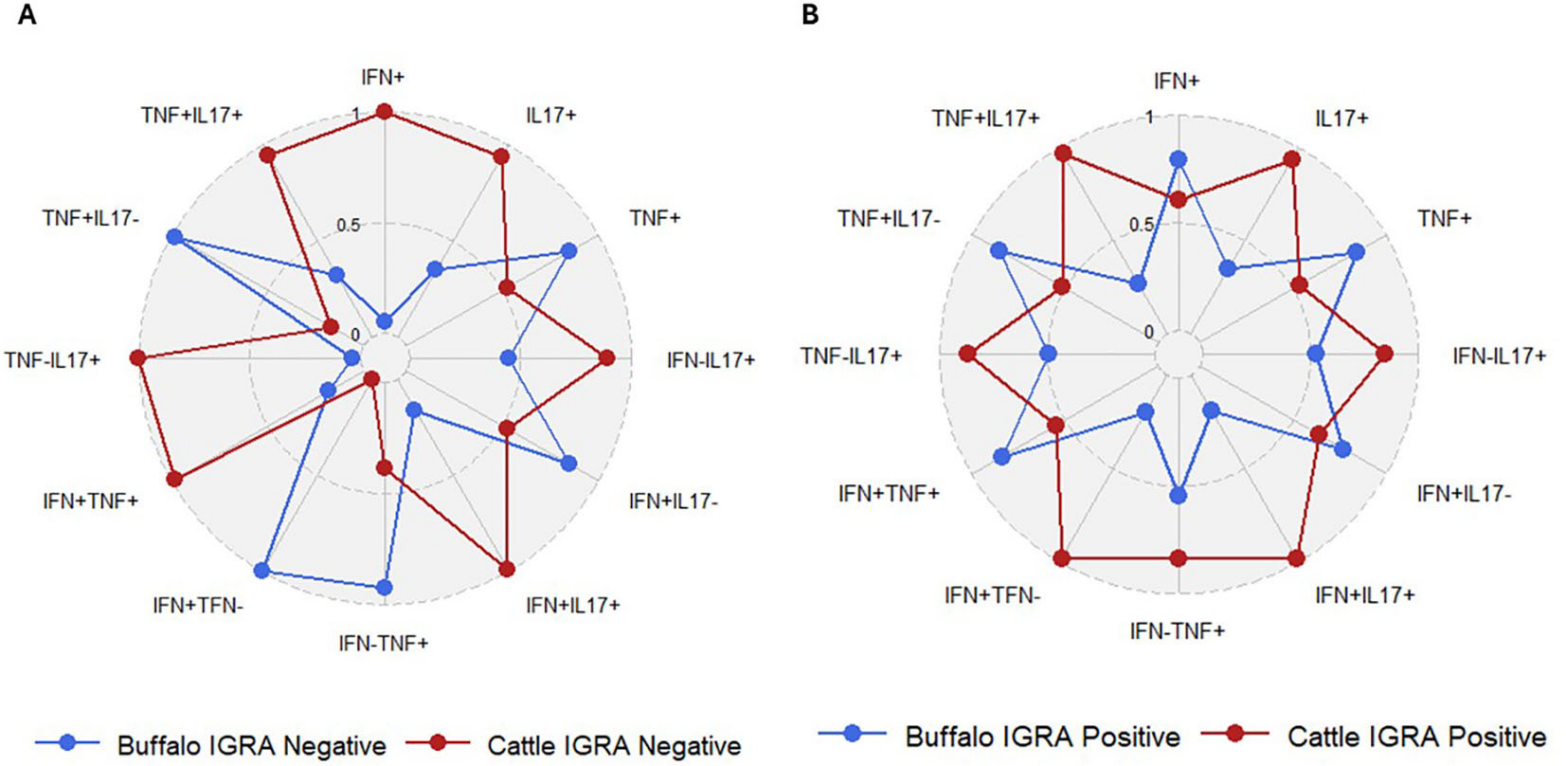
Spider plots based on data from **Figure 7**, showing the frequencies of defined single and dual cytokine-producing CD4^+^ T subsets in IGRA-negative animals **(A)** and IGRA-positive animals **(B)**.

To determine whether the frequencies of single and dual cytokine-producing CD4^+^ T cells were different between buffaloes and cattle a comparative analysis was conducted. No significant differences were observed between IGRA negative buffaloes and cattle (**Figure 7A**), nor between IGRA positive buffaloes and cattle (**Figure 7B**) (**Supplementary Table S4**). However, the spider plots reveal distinct differences in the frequencies of cytokine-positive CD4^+^ lymphocyte subsets between cattle and buffalo. Notably, subsets such as IFN-γ^+^IL-17A^+^, IFN-γ^neg^IL-17A^+^, TNF-α^+^IL-17A^+^, IL - 17A^+^ and TNF-α^neg^IL-17A^+^ within CD4^+^ T cells, are consistently more frequent in cattle than in buffalo, regardless of IGRA test status (**Figure 8**). These patterns highlight species-specific differences in immune response profiles.

As stated in the material & methods section, all animals (cattle and buffalo) were from farms with a BTB outbreak or from a OTF farm. However, we also analyzed a group of buffaloes negative to IGRA test from the BTB outbreak farm. Therefore, we classified the animals in three groups according to their IGRA test result, Group A: IGRA positive buffaloes from a BTB farm; Group B: IGRA negative buffaloes from a BTB farm; and Group C: IGRA test negative buffaloes from a OTF farm.

**Figure 9:**
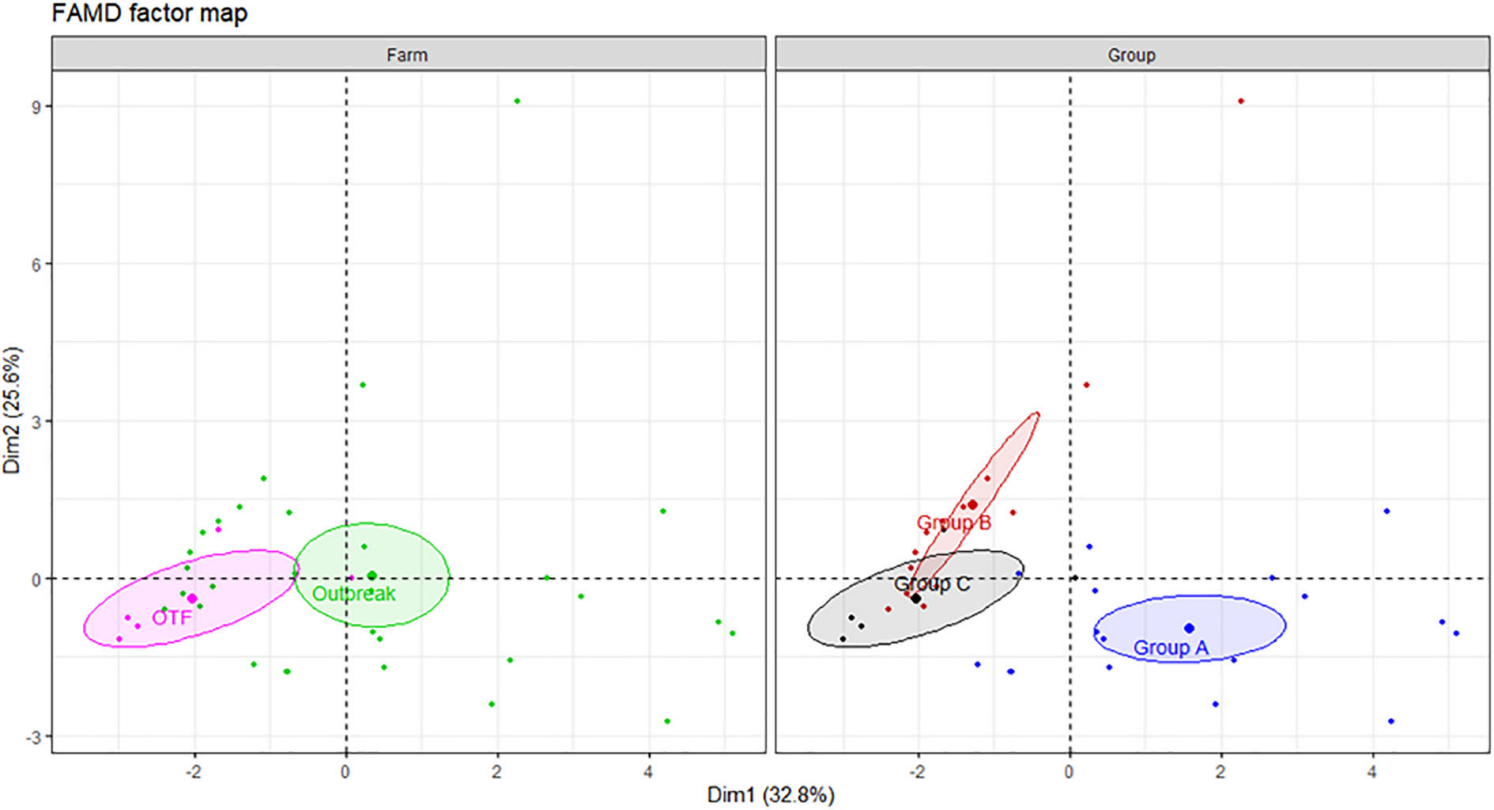
FAMD factor map based on single and dual cytokine-producing cells with CD4^+^ T cells in buffaloes from a BTB and OTF farms. Buffaloes were divided into three groups based on results of the IGRA test and the status of the herd. Group A) IGRA positive animals from BTB outbreak farm; Group B) IGRA negative animals from BTB outbreak farm; Group C) IGRA negative animals from OTF farm. The left panel shows clustering by farm (OTF in pink, BTB outbreak in green), and the right panel by group (group A in blue, group B in red, and group C in black). Ellipses represent 95% confidence intervals. Dim1 and Dim2 explain 32.8% and 25.6% of the variance, respectively, indicating distinct immunological profiles by farm and groups. A total of 17 IGRA-positive from BTB outbreak farm (group A), 13 IGRA-negative animals from BTB outbreak farm (group B) and 5 IGRA-negative animals from OTF farm (group C) were included in the analysis.

The FAMD analysis explain a combined variation of 58.4% (Dim1: 32.8% and Dim2: 25.6%, respectively). Dim1 is represented mostly by the contribution of IFN-γ^+^, TNF-α^+^, IFN-γ^+^TNF-α^+^, IFN-γ^+^IL-17A^neg^ and TNF-α^+^IL-17A^neg^ CD4^+^ cells. Whereas IL - 17A^+^, IFN-γ^neg^TNF-α^+^, IFN-γ^neg^IL-17A^+^, TNF-α^+^IL-17A^+^, TNF-α^neg^IL-17A^+^ CD4^+^ T cells have the highest contribution to Dim2 (**Supplementary Table S5**). The farm panel shows that there is a separation between BTB and OTF farms along Dim1, suggesting that this dimension captures key differences between farms. The right panel of groups revealed three distinct clustering patterns. Group A (IGRA positive animals) is well separated from Group B and C (IGRA negative animals), particularly along Dim1. Interestingly, Groups B and C formed distinct clusters with partial overlap in the center-left area of the plot, suggesting a partial immunological difference that may be related to the herd type (BTB outbreak vs. OTF) (**Figure 9**).

**Figure 10:**
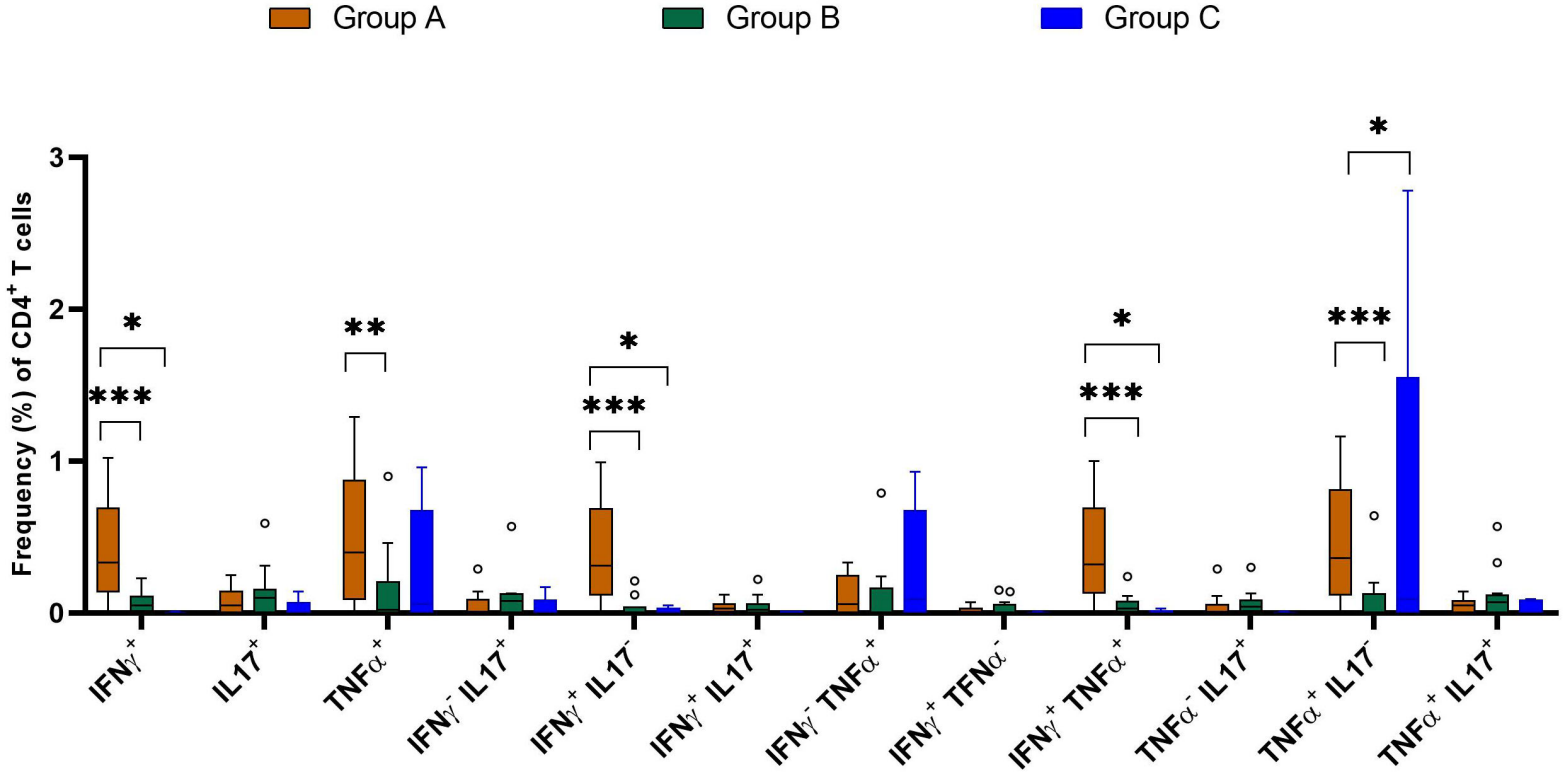
Frequencies (%) of single and dual cytokine-producing CD4^+^ T subsets in buffaloes from BTB (Group A and B) and OTF farms (Group C). Group A= IGRA-positive from BTB farm; Group B= IGRA-negative from BTB farm; and Group C= IGRA-negative from OTF farm. Frequencies (%) of reactive cells are displayed after subtraction of cells stimulated with PBS. A total of 17 IGRA-positive from BTB outbreak farm (group A), 13 IGRA-negative animals from BTB outbreak farm (group B) and 5 IGRA-negative animals from OTF farm (group C) were included in the analysis. Empty dots represent individual outliers. Results were compared using two-way ANOVA with Šidák multiple comparison test. *P < 0.05; **P < 0.001; ***P < 0.0001.

Further comparative analysis demonstrated significant higher cell frequencies of IFN-γ^+^, TNF-α^+^, IFN-γ^+^TNF-α^+^, IFN-γ^+^IL-17A^neg^ and TNF-α^+^IL-17A^neg^ CD4^+^ T subsets in the Group A compared with Groups B and C (**Figure 10**). The comparative analysis showed that the frequency of cytokine-positive CD4^+^ T lymphocytes was generally comparable among IGRA-negative buffaloes; however, a modest but statistically significant increase in TNF-α^+^ and TNF-α^+^IL-17A^-^ CD4^+^ T cell subsets was observed in animals from farm C compared to farm B (**Figure 9**). This finding may reflect farm-specific environmental or management factors influencing baseline immune responses.

When comparing IGRA-positive buffaloes (Group A) with IGRA-negative animals (Groups B and C), clear differences in cytokine-producing CD4^+^ T cell subsets were evident, consistent with the patterns of IGRA response (**Supplementary Figure S1**).

These results suggest that immune response variation is not solely a reflection of environmental exposure but likely indicates active immune engagement triggered by *M. bovis* infection, as marked by IGRA positivity. This supports the interpretation that functional immune activation, rather than passive pathogen exposure, underlies the observed cellular differences.

## 4 Discussion

Bovine tuberculosis (BTB) is a complex disease involving multiple host species, including humans, domestic and wild animals. In endemic regions, zoonotic tuberculosis cases are frequently reported, posing a significant public health concern (25). Additionally, BTB has a major economic impact on the livestock industry due to production losses, trade restrictions, and cost associated with national eradication programs (26). Despite extensive control efforts, BTB remains widespread across all continents except Antarctica, highlighting the need for interdisciplinary approaches to optimize disease management (27).

Buffaloes play a crucial role in livestock production, contributing to milk, meat, leather, and agricultural labor. Although they are susceptible to the same diseases as cattle, differences in physiology and behavior influence their disease susceptibility. For instance, buffaloes experience lower tick infestations than cattle (28), yet their wallowing behavior increases exposure to environmental pathogens, including parasites and non-tuberculous mycobacteria (29). Despite these known behavioral differences, the immunological mechanisms underlying the buffalo’s response to infectious diseases, including BTB, remain largely uncharacterized. The study herein addresses this gap in part by evaluating cytokine responses in buffaloes naturally exposed to BTB and comparing them to cattle with a particular focus on polyfunctional CD4^+^ T cell response.

Disease progression in BTB-infected buffaloes is generally slower than in cattle, which may be attributed to genetic and physiological factors (18, 30). One notable challenge in buffaloes is the difficulty in interpreting tuberculin skin test results due to their thicker skin and greater exposure to environmental *Mycobacteria*, which may interfere with diagnostic accuracy (31). Understanding species-specific immune responses is essential for improving diagnostic strategies and eradication efforts for both domestic and wildlife species.

This study aimed to assess an assay for detecting polyfunctional CD4^+^ T cells expressing IFN-γ, TNF-α, and IL - 17A in buffaloes naturally exposed to *M. bovis*. By analyzing the frequency of single and dual cytokine-producing CD4^+^ T subsets, we sought to compare immune responses between buffaloes and cattle. Previous studies have shown that different commercial anti-bovine mAbs are cross-reactive to buffalo leukocytes (19, 23). Our findings confirm that the anti-bovine monoclonal antibodies used in this study effectively cross-react with buffalo cells. This technical validation is crucial for future research into immune responses in buffaloes, a species often overlooked in TB studies.

In this study, we observed a higher frequency of antigen-specific IFN-γ-producing CD4^+^ lymphocytes in IGRA-positive buffaloes compared to IGRA-negative animals, reinforcing the association between IFN-γ responses and *M. bovis* infection status. This aligns with the well-established role of IFN-γ in TB immunity, where CD4^+^ T cells contribute to bacterial control and host survival during both acute and chronic infections. These findings are consistent with our previous work (19), which demonstrated a significant increase in IFN-γ^+^ CD4^+^ T cells in infected buffaloes compared to uninfected animals across different stimuli. This reinforces the utility of IFN-γ responses as a reliable immunological marker in BTB-endemic settings, not only in cattle but also in buffaloes.

Similarly, IGRA-positive cattle exhibited increased IFN-γ-producing CD4^+^ T lymphocytes, although differences compared to IGRA-negative animals were not statistically significant. It seems that this is related to sample size or individual variability in immune responses. Nevertheless, given that a high frequency of IFN-γ producing CD4^+^ T lymphocytes is a hallmark of active TB, our results suggest that this marker remains relevant for both buffaloes and cattle in BTB-endemic settings (32).

TNF-α is another cytokine that plays a crucial role in controlling mycobacterial infections, particularly during chronic TB. A previous study reported higher levels of PPD-B-specific TNF-α in infected animals compared to uninfected ones (33). In contrast, in the present study of naturally exposed buffaloes, we did not observe significant differences in TNF-α expression between IGRA-positive and IGRA-negative animals. This discrepancy may be attributed to differences in stimulation protocols—24 hours for ELISA assays in the previous study versus six hours for flow cytometry here—or to the distinct sources of TNF-α measured (plasma versus intracellular production by CD4^+^ T cells). Another difference may be due to the timing of infection, as early stages may not yet trigger strong TNF-α responses.

In this study, it is important to recognize that only one buffalo originating from a herd with confirmed *M. bovis* infection, was classified as infected based on the presence of macroscopic lesions and PCR positivity. The remaining buffaloes, although were IGRA-positive from BTB-affected herds, showed no visible lesions or bacteriological confirmation. This likely reflects early stages of infection, which may influence cytokine profiles. In contrast, cattle with visible lesions exhibited higher cytokine levels among IGRA-positive animals, although these differences were not statistically significant, potentially due to sample size limitations (33).

These findings underscore the challenges of distinguishing infection stages using current diagnostic tools and highlight the need to interpret cytokine responses cautiously, particularly when definitive confirmation of infection is lacking.

In line with established BTB control programs, animals from infected herds that test positive by IGRA are routinely considered infected, despite known limitations in diagnostic sensitivity and specificity. It is worth noting that IGRA can detect infection as early as 15 days post-exposure, a phase in which macroscopic lesions are generally absent and bacteriological confirmation is difficult (34). However, the possibility of nonspecific immune responses in IGRA-positive animals cannot be entirely excluded. As a result, interpreting immune profiles based solely on IGRA status may introduce bias, especially if immune responses differ between recent exposure and established infection. This limitation should be considered when evaluating immunological data and disease progression in naturally exposed populations.

In humans, elevated frequencies of TNF-α-producing CD4^+^ T cells have been associated with active disease stages (35), and increased IFN-γ^+^TNF-α^+^ CD4^+^ T lymphocytes have been reported in TB patients compared to healthy household contacts (36). Similar findings have also been observed on naturally infected cattle (12). Our results align with these findings, demonstrating a higher proportion of IFN-γ^+^TNF-α^+^ co-producing CD4^+^ T cells in animals classified as IGRA-positive. This suggests that the simultaneous expression of IFN-γ and TNF-α, rather than TNF-α production alone, may provide useful insight into immune activation and potentially help distinguish disease stages in ruminants. However, it is important to note that the concept of latent TB in cattle and buffaloes remains controversial, largely due to the lack of reliable, stage-specific diagnostic markers (37). Current diagnostic tools do not differentiate between infection stages, resulting in the culling of all test-positive animals (38). In this context, flow cytometry-based approaches capable of evaluating polyfunctional T cell responses could offer more refined diagnostic insights and support improved disease management strategies (39).

Additionally, IL-17A and Th17 responses are known to contribute to protective immunity against TB (40). Infected cattle and buffaloes have been shown to produce higher levels of PPD-B-specific IL-17A compared to uninfected animals (14, 33) it was reported that IFN-γ and IL-17A synergistically enhance intracellular mycobacterial control by promoting phagolysosomal fusion (41). Further, dual producing IFN-γ^+^IL-17A^+^ CD4^+^ T cells have been identified as key markers of balanced immune responses in TB vaccine studies (41). However, in our study, we did not detect significant differences in IL-17A-producing CD4^+^ T cells between exposed and unexposed animals. These results suggest that while IL-17A plays a role in TB immunity, its cellular sources and dynamics during natural BTB infection warrant further investigation.

When comparing buffalo and cattle, overall frequencies of CD4^+^ T cells did not differ significantly between species. However, certain cytokine-expressing subsets were more prevalent in cattle than in buffaloes, particularly in IGRA positive animals. This could reflect intrinsic differences in immune responses or variations in disease progression. Future studies should include larger sample sizes, particularly for cattle, to determine whether species-specific immune profiles exist.

In addition, the results from the FAMD analysis offer valuable insights into the differentiation of buffalo groups based on their IGRA test status and farm origin. The separation of IGRA-positive animals observed in the FAMD analysis highlights distinct immunological or physiological profiles that may reflect different stages of immune activation or exposure to *M. bovis*. When analyzing the three groups of buffaloes based on IGRA status and farm origin, clear immunological differences were evident. IGRA-positive buffaloes from BTB-infected farms (Group A) displayed significantly higher frequencies of IFN-γ^+^, TNF-α^+^ and IFN-γ^+^TNF-α^+^ polyfunctional CD4^+^ T cell subsets compared to both IGRA-negative groups. Interestingly, although overall cytokine expression among IGRA-negative animals was comparable, buffaloes from the OTF herd (Group C) exhibited slightly elevated TNF-α^+^ and TNF-α^+^IL-17A^neg^ CD4^+^ T cell subsets relative to IGRA-negative buffaloes from the BTB farm (Group B). This subtle variation suggests that environmental or farm-specific factors may influence baseline immune profiles, even in uninfected animals. However, the marked increase in cytokine-producing CD4^+^ T cells in IGRA-positive animals reinforces the association between functional T cell activation and likely *M. bovis* exposure.

In conclusion, our study introduced a novel approach for detecting cytokine co-expression in CD4^+^ T lymphocytes, providing new insights into the immune response of buffaloes and cattle naturally exposed to BTB. Notably, specific polyfunctional CD4^+^ T cell subsets, particularly IFN-γ^+^TNF-α^+^, IFN-γ^+^IL-17A^neg^, and other IFN-γ–associated profiles, correlated with IGRA status, distinguishing exposed from unexposed animals. These findings highlight the potential of IFN-γ–based T cell profiling to complement existing BTB diagnostic strategies in ruminants.

However, it is important to interpret these results considering the challenges in confirming infection status, as animals from BTB-infected herds may represent different stages of infection, and nonspecific IGRA responses cannot be entirely ruled out. Nonetheless, our data support the utility of multiparametric flow cytometry as a sensitive tool for characterizing complex cellular immune responses in comparative veterinary immunology.

These insights contribute to understanding BTB pathogenesis and may guide the development of improved diagnostic tools, vaccines, and tailored control strategies. In particular, identifying polyfunctional CD4^+^ T cell subsets co-expressing IFN-γ and TNF-α offers promising biomarker candidates to enhance diagnostic specificity, especially for distinguishing between exposure and active infection stages. Moreover, recognizing species-specific immune variations provides a basis for designing more effective, targeted vaccines and refining BTB management approaches according to the distinct disease dynamics in buffaloes and cattle. However, further research is needed to confirm the functional significance of these immune responses, clarify their role in disease monitoring, and explore their potential application in vaccine development, including investigations of additional T cell subsets and immune markers. Such advances may ultimately contribute to more effective BTB control and eradication efforts across species.

## Data Availability

The original contributions presented in the study are included in the article/**Supplementary Material**. Further inquiries can be directed to the corresponding author.

## References

[1] Cousins DV. Mycobacterium bovis infection and control in domestic livestock. Rev Sci Tech (International Office Epizootics). (2001) 20:71–85. doi: 10.20506/rst.20.1.1263, PMID: 11288521

[2] Gortázar C Che Amat A O’Brien DJ. Open questions and recent advances in the control of a multi-host infectious disease: animal tuberculosis. Mammal Rev. (2015) 45:160–75. doi: 10.1111/mam.12042

[3] Olea-Popelka F Muwonge A Perera A Dean AS Mumford E Erlacher-Vindel E. Zoonotic tuberculosis in human beings caused by Mycobacterium bovis-a call for action. Lancet Infect Dis. (2017) 17:e21–e5. doi: 10.1016/s1473-3099(16)30139-6, PMID: 27697390

[4] Bertoni A Napolitano F Mota-Rojas D Sabia E Álvarez-Macías A Mora-Medina P. Similarities and differences between river buffaloes and cattle: health, physiological, behavioral and productivity aspects. J Buffalo Sci. (2020) 9:92–109. doi: 10.6000/1927-520X.2020.09.12

[5] El-Ashker M Gwida M Monecke S El-Gohary F Ehricht R Elsayed M. Antimicrobial resistance pattern and virulence profile of S. aureus isolated from household cattle and buffalo with mastitis in Egypt. Vet Microbiol. (2020) 240:108535. doi: 10.1016/j.vetmic.2019.108535, PMID: 31902507

[6] Motta Giraldo JL Waltero García I Abeledo García MA Miranda I R. CP. Principales transtornos reproductivos en búfalas y vacas en hatos mixtos y de una especie en el departamente de Caquetá. Colombia Rev Fac Med Vet Zoot. (2014) 61:228–40. doi: 10.15446/rfmvz.v61n3.46870

[7] Mayer-Barber KD Barber DL. Innate and adaptive cellular immune responses to mycobacterium tuberculosis infection. Cold Spring Harbor Perspect Med. (2015) 5. doi: 10.1101/cshperspect.a018424, PMID: 26187873 PMC4665043

[8] O’Garra A Redford PS McNab FW Bloom CI Wilkinson RJ Berry MP. The immune response in tuberculosis. Annu Rev Immunol. (2013) 31:475–527. doi: 10.1146/annurev-immunol-032712-095939, PMID: 23516984

[9] Morgan J Muskat K Tippalagama R Sette A Burel J Lindestam Arlehamn CS. Classical CD4 T cells as the cornerstone of antimycobacterial immunity. Immunol Rev. (2021) 301:10–29. doi: 10.1111/imr.12963, PMID: 33751597 PMC8252593

[10] Caccamo N Guggino G Joosten SA Gelsomino G Di Carlo P Titone L. Multifunctional CD4+ T cells correlate with active Mycobacterium tuberculosis infection. Eur J Immunol. (2010) 40:2211–20. doi: 10.1002/eji.201040455, PMID: 20540114

[11] Day CL Abrahams DA Lerumo L Janse van Rensburg E Stone L O’Rie T. Functional capacity of Mycobacterium tuberculosis-specific T cell responses in humans is associated with mycobacterial load. J Immunol (Baltimore Md: 1950). (2011) 187:2222–32. doi: 10.4049/jimmunol.1101122, PMID: 21775682 PMC3159795

[12] Whelan AO Villarreal-Ramos B Vordermeier HM Hogarth PJ. Development of an antibody to bovine IL - 2 reveals multifunctional CD4 T(EM) cells in cattle naturally infected with bovine tuberculosis. PloS One. (2011) 6:e29194. doi: 10.1371/journal.pone.0029194, PMID: 22216206 PMC3245252

[13] Lewinsohn DA Lewinsohn DM Scriba TJ. Polyfunctional CD4(+) T cells as targets for tuberculosis vaccination. Front Immunol. (2017) 8:1262. doi: 10.3389/fimmu.2017.01262, PMID: 29051764 PMC5633696

[14] Maggioli MF Palmer MV Thacker TC Vordermeier HM McGill JL Whelan AO. Increased TNF-α/IFN-γ/IL-2 and decreased TNF-α/IFN-γ Production by central memory T cells are associated with protective responses against bovine tuberculosis following BCG vaccination. Front Immunol. (2016) 7:421. doi: 10.3389/fimmu.2016.00421, PMID: 27799930 PMC5066095

[15] Steinbach S Vordermeier HM Jones GJ. CD4+ and γδ T Cells are the main Producers of IL - 22 and IL - 17A in Lymphocytes from Mycobacterium bovis-infected Cattle. Sci Rep. (2016) 6:29990. doi: 10.1038/srep29990, PMID: 27427303 PMC4947955

[16] Elnaggar MM Abdellrazeq GS Dassanayake RP Fry LM Hulubei V Davis WC. Characterization of αβ and γδ T cell subsets expressing IL - 17A in ruminants and swine. Dev Comp Immunol. (2018) 85:115–24. doi: 10.1016/j.dci.2018.04.003, PMID: 29627456

[17] Minervino AHH Zava M Vecchio D Borghese A. Bubalus bubalis: A short story. Front Vet Sci. (2020) 7:570413. doi: 10.3389/fvets.2020.570413, PMID: 33335917 PMC7736047

[18] Carneiro PAM Takatani H Pasquatti TN Silva C Norby B Wilkins MJ. Epidemiological study of mycobacterium bovis infection in buffalo and cattle in amazonas, Brazil. Front Vet Sci. (2019) 6:434. doi: 10.3389/fvets.2019.00434, PMID: 31921899 PMC6914675

[19] De Matteis G Scatà MC Zampieri M Grandoni F Elnaggar MM Schiavo L. Flow cytometric detection of IFN-γ production and Caspase-3 activation in CD4(+) T lymphocytes to discriminate between healthy and Mycobacterium bovis naturally infected water buffaloes. Tuberculosis (Edinburgh Scotland). (2023) 139:102327. doi: 10.1016/j.tube.2023.102327, PMID: 36857964

[20] Elnaggar MM El-Naggar MM Abdellrazeq GS Sester M Khaliel SA Singh M. Development of an improved ESAT - 6 and CFP - 10 peptide-based cytokine flow cytometric assay for bovine tuberculosis. Comp Immunol Microbiol Infect Dis. (2015) 42:1–7. doi: 10.1016/j.cimid.2015.07.005, PMID: 26577191

[21] WOAH. Chapter 3.1.13 - Mammalian tuberculosis: Infection with Mycobacterium tuberculosis complex. In: Terrestrial Animal Health Manual (2022). Available online at: https://www.woah.org/en/what-we-do/animal-health-and-welfare/disease-data-collection/.

[22] Martucciello A Vitale N Mazzone P Dondo A Archetti I Chiavacci L. Field evaluation of the interferon gamma assay for diagnosis of tuberculosis in water buffalo (Bubalus bubalis) comparing four interpretative criteria. Front Vet Sci. (2020) 7:563792. doi: 10.3389/fvets.2020.563792, PMID: 33335916 PMC7736034

[23] Grandoni F Elnaggar MM Abdellrazeq GS Signorelli F Fry LM Marchitelli C. Characterization of leukocyte subsets in buffalo (Bubalus bubalis) with cross-reactive monoclonal antibodies specific for bovine MHC class I and class II molecules and leukocyte differentiation molecules. Dev Comp Immunol. (2017) 74:101–9. doi: 10.1016/j.dci.2017.04.013, PMID: 28433527

[24] Pagès J. Analyse factorielle de données mixtes. Rev Statistique Appliquée. (2004) 52:93–111. doi: 10.4236/am.2018.98065

[25] Torres-Gonzalez P Cervera-Hernandez ME Martinez-Gamboa A Garcia-Garcia L Cruz-Hervert LP Bobadilla-Del Valle M. Human tuberculosis caused by Mycobacterium bovis: a retrospective comparison with Mycobacterium tuberculosis in a Mexican tertiary care centre, 2000 - 2015. BMC Infect Dis. (2016) 16:657. doi: 10.1186/s12879-016-2001-5, PMID: 27825312 PMC5101666

[26] Conteddu K English HM Byrne AW Amin B Griffin LL Kaur P. A scoping review on bovine tuberculosis highlights the need for novel data streams and analytical approaches to curb zoonotic diseases. Vet Res. (2024) 55:64. doi: 10.1186/s13567-024-01314-w, PMID: 38773649 PMC11110237

[27] Ramos B Pereira AC Reis AC Cunha MV. Estimates of the global and continental burden of animal tuberculosis in key livestock species worldwide: A meta-analysis study. One Health (Amsterdam Netherlands). (2020) 10:100169. doi: 10.1016/j.onehlt.2020.100169, PMID: 33134472 PMC7582805

[28] Batista HR Sarturi C Stelmachtchuk FN Oliveira DR Morini AC Gennari SM. Prevalence and risk factors associated with ectoparasite infestation of buffaloes in an Amazonian ecosystem. Parasites Vectors. (2018) 11:335. doi: 10.1186/s13071-018-2917-2, PMID: 29866180 PMC5987401

[29] Aranganoor Kannan T Hussain T Avendaño-Reyes L Ramanujam R. Editorial: Buffalo (swamp and riverine) production for meat and milk. Front Anim Sci. (2023) 4:1284368. doi: 10.3389/fanim.2023.1284368

[30] Iannaccone M Cosenza G Pauciullo A Martino C Ianni A Capparelli R. Water buffalo (Bubalus bubalis) susceptibility to bovine tuberculosis is influenced by g.4002c>t polymorphism in interleukin-10 gene. Buffalo Bull. (2019) 38:135–9.

[31] Martucciello A Ottaiano M Mazzone P Vitale N Donniacuo A Brunetti R. A decade of tuberculosis eradication programs in the Mediterranean water buffalo (Bubalus bubalis) in South Italy: Are we heading toward eradication? Front Vet Sci. (2024) 11:1405416. doi: 10.3389/fvets.2024.1405416, PMID: 39132442 PMC11310139

[32] Boggiatto PM Kanipe CR Palmer MV. Enhanced detection of mycobacterium bovis-specific T cells in experimentally-infected cattle. Front Vet Sci. (2021) 8:676710. doi: 10.3389/fvets.2021.676710, PMID: 34336973 PMC8317970

[33] Franzoni G Signorelli F Mazzone P Donniacuo A De Matteis G Grandoni F. Cytokines as potential biomarkers for the diagnosis of Mycobacterium bovis infection in Mediterranean buffaloes (Bubalus bubalis). Front Vet Sci. (2024) 11:1512571. doi: 10.3389/fvets.2024.1512571, PMID: 39776597 PMC11703857

[34] Buddle BM de Lisle GW Pfeffer A Aldwell FE. Immunological responses and protection against Mycobacterium bovis in calves vaccinated with a low dose of BCG. Vaccine. (1995) 13:1123–30. doi: 10.1016/0264-410x(94)00055-r, PMID: 7491820

[35] Harari A Rozot V Enders FB Perreau M Stalder JM Nicod LP. Dominant TNF-α+ Mycobacterium tuberculosis–specific CD4+ T cell responses discriminate between latent infection and active disease. Nat Med. (2011) 17:372–6. doi: 10.1038/nm.2299, PMID: 21336285 PMC6570988

[36] Sutherland JS Adetifa IM Hill PC Adegbola RA Ota MO. Pattern and diversity of cytokine production differentiates between Mycobacterium tuberculosis infection and disease. Eur J Immunol. (2009) 39:723–9. doi: 10.1002/eji.200838693, PMID: 19224636

[37] Sabio YGJ Bigi MM Klepp LI García EA Blanco FC Bigi F. Does Mycobacterium bovis persist in cattle in a non-replicative latent state as Mycobacterium tuberculosis in human beings? Vet Microbiol. (2020) 247:108758. doi: 10.1016/j.vetmic.2020.108758, PMID: 32768211

[38] Lahuerta-Marin A Gallagher M McBride S Skuce R Menzies F McNair J. Should they stay, or should they go? Relative future risk of bovine tuberculosis for interferon-gamma test-positive cattle left on farms. Vet Res. (2015) 46:90. doi: 10.1186/s13567-015-0242-8, PMID: 26338808 PMC4559371

[39] Ncube P Bagheri B Goosen WJ Miller MA Sampson SL. Evidence, challenges, and knowledge gaps regarding latent tuberculosis in animals. Microorganisms. (2022) 10. doi: 10.3390/microorganisms10091845, PMID: 36144447 PMC9503773

[40] Waters WR Maggioli MF Palmer MV Thacker TC McGill JL Vordermeier HM. Interleukin-17A as a biomarker for bovine tuberculosis. Clin Vaccine Immunol: CVI. (2016) 23:168–80. doi: 10.1128/cvi.00637-15, PMID: 26677202 PMC4744917

[41] Choi HG Kwon KW Choi S Back YW Park HS Kang SM. Antigen-specific IFN-γ/IL-17-co-producing CD4(+) T-cells are the determinants for protective efficacy of tuberculosis subunit vaccine. Vaccines. (2020) 8. doi: 10.3390/vaccines8020300, PMID: 32545304 PMC7350228

