# Supplementary material for: Polyfunctionality of CD4+ T lymphocytes in buffaloes and cattle: comparative antigen-specific cytokine responses in bovine tuberculosis infection
Source: Front Immunol. 2025 Sep 8;16:1608065. doi: 10.3389/fimmu.2025.1608065 (PMC12450747; doi:10.3389/fimmu.2025.1608065)
Supplement: Supplementary file 1 [file DataSheet1.docx]

Supplementary Tables and Figures

| **Specificity** | **mAb clone** | **Species reactivity** | **Source** | **Labelling** | **Reference** |
| --- | --- | --- | --- | --- | --- |
| TNF-α | TNF-α | Bovine | WSUMAC, USA* | FITC** | this study *** |
| IL-17A | IL-17A | Bovine, | WSUMAC, USA* | PE** | this study *** |
| CD4 | ILA11A | Bovine | WSUMAC, USA* | PE-CY7** | Grandoni et al., 2017 |
| IFN-γ | CC302 | Bovine | Bio-Rad | AF647 | De Matteis et al., 2023 |

**Supplementary Table 1.** List of monoclonal antibodies used for the four-color panel

******* *WSU-MAC = Washington State University-Monoclonal Antibody Center, Pullman, WA, USA*

******** *In house labelling using Lynx technology (Bio-Rad)*

********* *Referred to buffalo reactivity*

| Supplementary Table 2. Percentage contribution of the quantitative variables to the first two dimensions (Dim 1 and Dim 2) in the FAMD from single and dual-producing CD4^+^ T subsets in buffalo and cattle. | | |
| --- | --- | --- |
| Cell subset (quantitative variable) | **Dim.1** | **Dim 2** |
| IFN-γ^+^ | **15.8915526** | 3.2029524 |
| IL17A^+^ | 5.865177 | **18.8359034** |
| TNF-α^+^ | **15.1071389** | 0.309226 |
| IFN-γ^+^TNF-α^+^ | **15.4520981** | 2.2729484 |
| IFN-γ^+^IL17A^+^ | 3.0310383 | **15.1192372** |
| IFN-γ^+^TNF-α^neg^ | 0.725137 | 1.4085751 |
| IFN-γ^+^IL17A^neg^ | **10.6487193** | **9.4573759** |
| IFN-γ^neg^TNF-α^+^ | 2.81907 | **7.5270661** |
| IFN-γ^neg^ IL17A^+^ | 3.6267591 | 1.9025526 |
| TNF-α^+^IL17A^+^ | 3.5519198 | **21.2402364** |
| TNF-α^+^IL17A^neg^ | **5.9296692** | 0.1143979 |
| TNF-α^neg^IL17A^+^ | 2.0383218 | 2.0253709 |
| *Bold numbers highlighting the variables with principal contributions | | |

| Supplementary Table 3. Mean frequencies of single and dual-producing CD4^+^ T subsets analyzed in IGRA negative and IGRA positive animals. Results were compared using Wilcoxon test with Bonferroni correction. | | | | | |
| --- | --- | --- | --- | --- | --- |
| Buffaloes | | | | | |
|  | **IGRA-negative** | | **IGRA-positive** | |  |
| Cell subset | **Mean** | **SD** | **Mean** | **SD** | **P-adjusted** |
| IFN-γ^+^ | 0.002 | 0.004472 | 0.4359 | 0.3513 | 0.00439 |
| IL17A^+^ | 0.028 | 0.06261 | 0.07412 | 0.07649 | 0.113 |
| TNF-α^+^ | 0.282 | 0.4122 | 0.5118 | 0.4384 | 0.306 |
| IFN-γ^+^TNF-α^+^ | 0.006 | 0.01342 | 0.4318 | 0.3477 | 0.00439 |
| IFN-γ^+^IL17A^+^ | 0.004 | 0.005477 | 0.03824 | 0.03414 | 0.0376 |
| IFN-γ^+^TFN-α^neg^ | 0.002 | 0.004472 | 0.01824 | 0.02325 | 0.134 |
| IFN-γ^+^IL17A^neg^ | 0.012 | 0.02168 | 0.4165 | 0.3489 | 0.00412 |
| IFN-γ^neg^TNF-α^+^ | 0.288 | 0.3983 | 0.1035 | 0.1206 | 0.57 |
| IFN-γ^neg^IL17A^+^ | 0.034 | 0.07603 | 0.05 | 0.07706 | 0.392 |
| TNF-α^+^IL17A^+^ | 0.034 | 0.04669 | 0.04647 | 0.04513 | 0.57 |
| TNF-α^+^IL17A^neg^ | 0.638 | 1.205 | 0.4753 | 0.3913 | 0.307 |
| TNF-α^neg^IL17A^+^ | 0.002 | 0.004472 | 0.03824 | 0.07308 | 0.184 |
| Cattle | | | | | |
|  | **IGRA-negative** | | **IGRA-positive** | |  |
| Cell subset | **Mean** | **SD** | **Mean** | **SD** | **P-adjusted** |
| IFN-γ^+^ | 0.0375 | 0.06238 | 0.3333 | 0.2129 | 0.0666 |
| IL17A^+^ | 0.075 | 0.15 | 0.2 | 0.2489 | 0.471 |
| TNF-α^+^ | 0.175 | 0.3175 | 0.3267 | 0.4151 | 0.441 |
| IFN-γ^+^TNF-α^+^ | 0.0325 | 0.03403 | 0.28 | 0.2268 | 0.134 |
| IFN-γ^+^IL17A^+^ | 0.025 | 0.01915 | 0.2 | 0.2903 | 0.826 |
| IFN-γ^+^TFN-α^neg^ | 0 | 0 | 0.09167 | 0.1383 | 0.149 |
| IFN-γ^+^IL17A^neg^ | 0.0075 | 0.015 | 0.3467 | 0.4061 | 0.139 |
| IFN-γ^neg^TNF-α^+^ | 0.12 | 0.2142 | 0.1583 | 0.1891 | 0.909 |
| IFN-γ^neg^IL17A^+^ | 0.0675 | 0.135 | 0.08 | 0.1068 | 0.631 |
| TNF-α^+^IL17A^+^ | 0.1 | 0.1538 | 0.165 | 0.237 | 0.914 |
| TNF-α^+^IL17A^neg^ | 0.1075 | 0.215 | 0.2867 | 0.2572 | 0.185 |
| TNF-α^neg^IL17A^+^ | 0.06 | 0.12 | 0.06667 | 0.05854 | 0.57 |

| Supplementary Table 4. Mean frequencies of single and dual-producing CD4^+^ T subsets analyzed in buffalo and cattle. Results were compared using Wilcoxon test with Bonferroni correction. | | | | | |
| --- | --- | --- | --- | --- | --- |
| IGRA-negative | | | | | |
|  | **Buffalo** | | **Cattle** | |  |
| Cell subset | **Mean** | **SD** | **Mean** | **SD** | **P-adjusted** |
| IFN-γ^+^ | 0.002 | 0.004472 | 0.0375 | 0.06238 | 0.599 |
| IL17A^+^ | 0.028 | 0.06261 | 0.075 | 0.15 | 0.668 |
| TNF-α^+^ | 0.282 | 0.4122 | 0.175 | 0.3175 | 0.307 |
| IFN-γ^+^TNF-α^+^ | 0.006 | 0.01342 | 0.0325 | 0.03403 | 0.344 |
| IFN-γ^+^IL17A^+^ | 0.004 | 0.005477 | 0.025 | 0.01915 | 0.696 |
| IFN-γ^+^TFN-α^neg^ | 0.002 | 0.004472 | 0 | 0 | 0.528 |
| IFN-γ^+^IL17A^neg^ | 0.012 | 0.02168 | 0.0075 | 0.015 | 0.649 |
| TNF-α^+^IL17A^+^ | 0.034 | 0.04669 | 0.1 | 0.1538 | 0.745 |
| IFN-γ^neg^TNF-α^+^ | 0.288 | 0.3983 | 0.12 | 0.2142 | 0.77 |
| IFN-γ^neg^IL17A^+^ | 0.034 | 0.07603 | 0.0675 | 0.135 | 0.738 |
| TNF-α^+^IL17A^neg^ | 0.638 | 1.205 | 0.1075 | 0.215 | 0.344 |
| TNF-α^neg^IL17A^+^ | 0.002 | 0.004472 | 0.06 | 0.12 | 0.273 |
| IGRA-positive | | | | | |
|  | **Buffalo** | | **Cattle** | |  |
| Cell subset | **Mean** | **SD** | **Mean** | **SD** | **P-adjusted** |
| IFN-γ^+^ | 0.4359 | 0.3513 | 0.3333 | 0.2129 | 0.308 |
| IL17A^+^ | 0.07412 | 0.07649 | 0.2 | 0.2489 | 0.123 |
| TNF-α^+^ | 0.5118 | 0.4384 | 0.3267 | 0.4151 | 0.771 |
| IFN-γ^+^TNF-α^+^ | 0.4318 | 0.3477 | 0.28 | 0.2268 | 0.701 |
| IFN-γ^+^IL17A^+^ | 0.03824 | 0.03414 | 0.2 | 0.2903 | 0.867 |
| IFN-γ^+^TFN-α^neg^ | 0.01824 | 0.02325 | 0.09167 | 0.1383 | 0.867 |
| IFN-γ^+^IL17A^neg^ | 0.4165 | 0.3489 | 0.3467 | 0.4061 | 0.178 |
| IFN-γ^neg^TNF-α^+^ | 0.1035 | 0.1206 | 0.1583 | 0.1891 | 0.701 |
| IFN-γ^neg^IL17A^+^ | 0.05 | 0.07706 | 0.08 | 0.1068 | 0.502 |
| TNF-α^+^IL17A^+^ | 0.04647 | 0.04513 | 0.165 | 0.237 | 0.867 |
| TNF-α^+^IL17A^neg^ | 0.4753 | 0.3913 | 0.2867 | 0.2572 | 0.502 |
| TNF-α^neg^IL17A^+^ | 0.03824 | 0.07308 | 0.06667 | 0.05854 | 0.383 |

| Supplementary Table 5. Percentage contribution of the quantitative variables to the first two dimensions (Dim 1 and Dim 2) in the FAMD for buffalos from BTB (Groups A and B) and OTF farms (Group C). | | |
| --- | --- | --- |
| Cell subset (quantitative variable) | **Dim.1** | **Dim 2** |
| IFN-γ^+^ | **16.08301** | 1.589696 |
| IL17A^+^ | 2.104732 | **20.07853** |
| TNF-α^+^ | **15.86134** | 0.2602008 |
| IFN-γ^+^TNF-α^+^ | **16.18963** | 1.753707 |
| IFN-γ^+^IL17A^+^ | 0.8935467 | 5.31354 |
| IFN-γ^+^TFN-α^neg^ | 0.2051749 | 0.6305273 |
| IFN-γ^+^IL17A^neg^ | **15.40622** | 2.586769 |
| IFN-γ^neg^TNFα^+^ | 2.132903 | **6.789421** |
| IFN-γ^neg^ IL17A^+^ | 2.05527 | **17.41014** |
| TNF-α^+^IL17A^+^ | 1.168347 | **18.43844** |
| TNF-α^+^IL17A^neg^ | **6.963603** | 0.02630611 |
| TNF-α^neg^IL17A^+^ | 1.061839 | **12.68368** |
| *Bold numbers highlighting the variables with principal contributions | | |


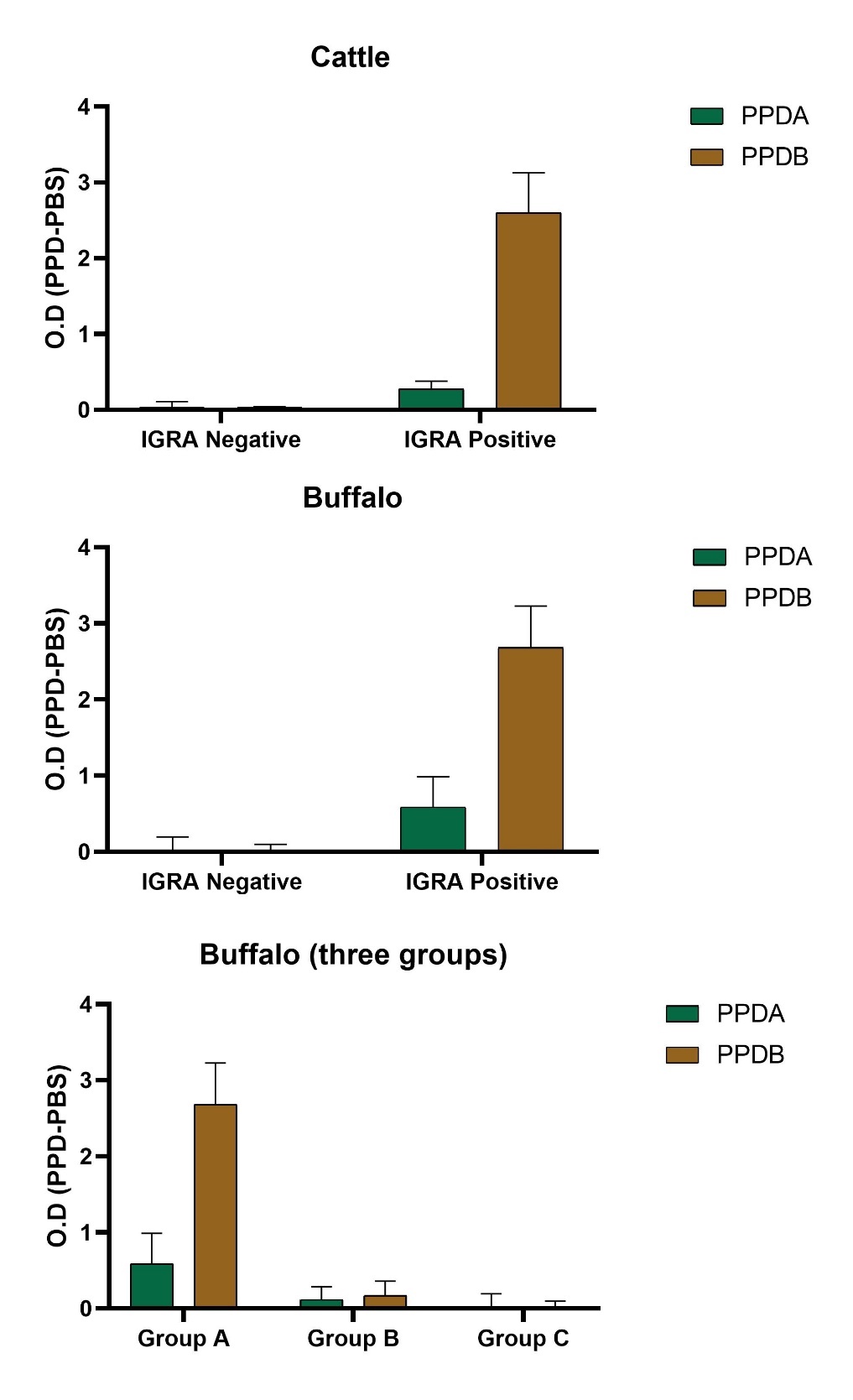


**Supplementary Figure 1**. IGRA results from all animals used in this study. The IGRA test was performed as described in material & methods. Results were used for the diagnosis of BTB disease and to classify animals as IGRA negative or IGRA positive. Data is presented as PPD-B minus PBS or PPD-A minus PBS. Cattle (n= 6 and 4, IGRA-positive and IGRA-negative, respectively). Buffalo (n= 17 and 5, IGRA-positive from BTB outbreak farm and IGRA-negative from OTF farm, respectively). Buffalo three groups: Group A) IGRA-positive animals from BTB outbreak farm (n=17); Group B) IGRA-negative animals from BTB outbreak farm (n=13); Group C) IGRA negative animals from OTF farm (n=5).

**
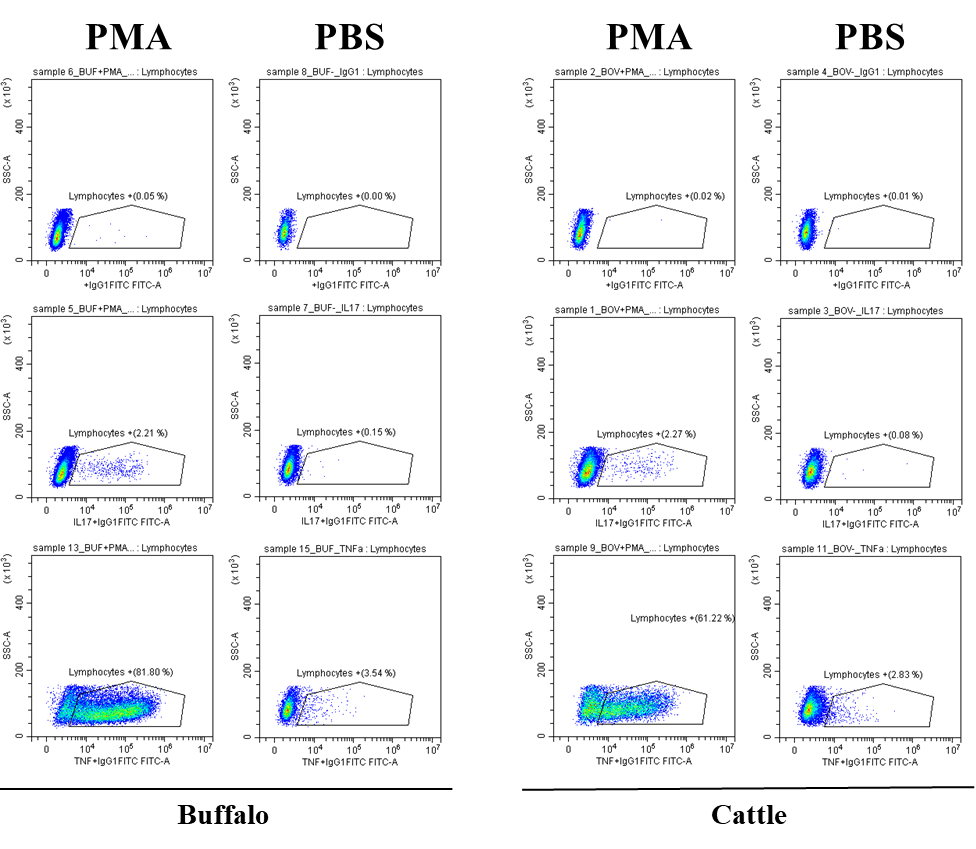
**

**Supplementary Figure 2.** Evaluation of the cross-reactivity of IL-17A (central dot plots) and TNF-α (bottom dot plots) monoclonal antibodies on buffalo PBMCs, compared to bovine labeling patterns. Negative control staining using only the anti-mouse IgG1-FITC secondary antibody is shown in the upper dot plots. PBMCs were stimulated (PMA) with Cell Stimulation Reagent containing Brefeldin A (Bio-Rad) for 4 hours; PBS-treated cells (PBS) were used as negative controls.

**Supplementary Figure 3**. Representative example of flow cytometric gating strategy, cytokine profiling, and FMO controls of CD4⁺ T cells in PWM-stimulated buffalo blood. (A) Time parameter vs SSC-A was used to exclude event bursts. (B) FSC-A vs FSC-H was applied to eliminate doublets and debris. (C) FSC-A vs SSC-A allowed for the identification of total lymphocytes. (D) CD4 PC7-A vs SSC-A was used to gate the CD4⁺ lymphocyte population. (E–G) Within the CD4⁺ subset, expression of IFN-γ, IL-17A, and TNF-α was analyzed. (J, M, P) Dual cytokine-producing cells within the CD4⁺ population were identified. Fluorescence Minus One (FMO) control was used for IFN-γ and IL-17A (H-I), for IFN-γ and TNF-α (K-L) and for TNF-α and IL-17A (N-O).

**Supplementary Figure 4**. Comparison of single and dual cytokine-producing CD4^+^ lymphocytes (IFN-γ^+^TNF-α^+^, IFN-γ^+^IL-17A^+^, TNF-α^+^IL-17A^+^) and CD4^+^ subsets (IFN-γ^+^TFN-α^neg^, IFN-γ^+^IL17A^neg^, IFN-γ^neg^TNF-α^+^, IFN-γ^neg^IL17A^+^, TNF-α^+^IL17A^neg^, TNF-α^neg^IL17A^+^) in buffalo and cattle after stimulation with PWM. Quadrants were drawn based on the Florescence Minus One control (FMO) for each cytokine in buffalo and in cattle.
